# Advancing in Sarcoma 3D Modeling: Identification of SRC as an Actionable Vulnerability in Doxorubicin‐Resistant Osteosarcoma Organoids

**DOI:** 10.1002/mco2.71134

**Published:** 2026-11-08

**Authors:** Borja Gallego, Dzohara Murillo, Carmen Huergo, Daniela Corte‐Torres, Andrea Ferreras, Iván Fernández‐Vega, Adela A. Fernández‐Velasco, Verónica Rey, Enol Álvarez‐González, Alejandro Braña, Jose Luis Llorente, Mara Santarelli, Benno Schwikowski, Mónica Álvarez‐Fernández, René Rodríguez

**Affiliations:** ^1^ Instituto De Investigación Sanitaria Del Principado de Asturias (ISPA) Oviedo Spain; ^2^ Instituto Universitario de Oncología del Principado de Asturias (IUOPA) Oviedo Spain; ^3^ Biobanco Del Principado de Asturias Oviedo Spain; ^4^ Servicio De Patología Hospital Universitario Central De Asturias Oviedo Spain; ^5^ Servicio De Traumatología Hospital Universitario Central De Asturias Oviedo Spain; ^6^ Servicio De Otorrinolaringología Hospital Universitario Central De Asturias Oviedo Spain; ^7^ CIBER En Oncología (CIBERONC) Madrid Spain; ^8^ Computational Systems Biomedicine Lab, Institut Pasteur Université Paris Cité Paris France; ^9^ Centro De Investigación del Cáncer CSIC‐Universidad De Salamanca Salamanca Spain; ^10^ Centro De Investigación en Nanomateriales y Nanotecnología (CINN) Consejo Superior de Investigaciones Científicas (CSIC) El Entrego Asturias Spain

**Keywords:** dasatinib, doxorubicin, drug resistance, organoids, sarcoma, SRC

## Abstract

Tumor organoids have emerged as the most promising avatar to model cancer and to be used as drug testing platform in personalized medicine protocols. The derivation of organoids from epithelial tumors is well established, and their use has proven useful for biomarker discovery, drug screening, and clinical decision support. However, generation of organoids from sarcomas is much less developed and current protocols require further optimization to fully harness their research and clinical potential. In this study, the authors examined the challenges of generating basement membrane extract‐based organoids from patient‐derived sarcoma samples. Out of 19 samples, 3D structures formed in seven (37%), but none could be propagated beyond the first passage. Alternatively, the authors established expandable organoids from sarcoma xenografts. From 14 xenograft samples, seven (50%) were successfully expanded over multiple passages and biobanked. Notably, xenografts from parental and doxorubicin‐resistant 143B osteosarcoma cells yielded long‐term cultures suitable for studying drug resistance. Transcriptomic and functional analyses revealed that SRC signaling contributes to doxorubicin resistance in osteosarcoma. Inhibiting SRC with dasatinib restored sensitivity to doxorubicin in vitro and in vivo by downregulating cJUN‐mediated ABCB1 expression. These findings support xenograft‐derived organoids as a viable platform for modeling sarcoma and investigating therapeutic resistance.

## Graphical abstract

3D organoids with long‐term growth potential can be successfully generated from sarcoma xenografts. Paired parental and doxorubicin‐resistant organoids constitute a novel 3D platform for investigating drug resistance in osteosarcoma. The authors showed that inhibition of SRC‐mediated signaling with dasatinib can reverse doxorubicin resistance in osteosarcoma by suppressing cJUN‐mediated ABCB1 expression.

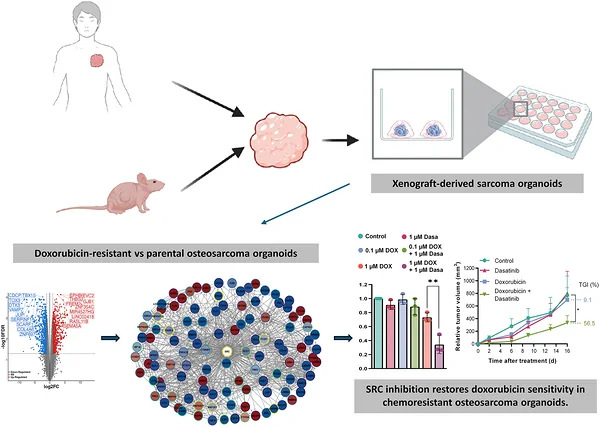

## 1 Introduction

Despite recent advances in sarcoma genomics and the clinical management of patients, systemic treatment for these tumors is still highly dependent on standard chemo and radiotherapy and around 80% of the advanced/metastatic cases still succumb to the disease due to emergence of therapy‐resistant clones [1, 2, 3, 4]. Having remained unchanged for the last decades, these data underscore the need for intensified research aimed at identifying novel therapeutic targets and developing improved preclinical models to predict drug response in functional personalized medicine approaches [1, 5, 6].

Among preclinical models, there is a growing consensus that tumor organoids have emerged as the most promising avatar to be used as drug testing platform of both standard‐of‐care and genomic‐informed therapies [7, 8]. By definition, these 3D models are derived from fresh tissue, exhibit self‐organizing capabilities, and their growth is driven by subpopulations of tumor cells with stem cell‐like properties (cancer stem cells). Therefore, this system supports the long‐term maintenance of structures that reproduce, at least in part, the complexity and genomics of the original tumors [7, 9]. Organoids can be generated using scaffold‐free and scaffold‐based protocols [10, 11, 12]. Scaffold‐free cultures typically rely on low‐adhesion surfaces and specific physical conditions. In contrast, scaffold‐based models utilize natural or synthetic materials to provide structural support and/or mimic the tumor microenvironment [11, 12, 13]. Among natural materials used as scaffolds, commercial hydrogels generated with basement membrane extracts (BME) of animal origin, such as Matrigel or Cultrex‐BME [14], are the most used. Regardless of the culture method, the media must be specifically tailored to meet the diverse growth requirements of tumor cells. This often includes growth factors, stemness pathway agonists, inhibitors of apoptosis and differentiation, and reactive oxygen species scavengers, among other components [12].

The use of organoids to model epithelial cancer types is well developed and has proven useful for the discovery of prognostic and predictive biomarkers, pharmacogenomics screens and clinical‐decision support [7, 15, 16, 17]. However, the generation of organoids from sarcomas is much less developed and only in recent years the derivation of scaffold free [18, 19, 20] and scaffold‐based models [12, 21, 22, 23, 24, 25] was reported. These models have proven their ability to recapitulate key features of the original tumors and were used to explore drug response profiles [21, 22, 23, 26], to correlate with patients’ clinical features [24] and to investigate determinants of cisplatin resistance [20, 27]. In any case, there are still technical requirements that need further refinement. For instance, some sarcoma models fail to form 3D structures in Matrigel [23] and/or have not demonstrated the capacity for long‐term cultivation, thus avoiding the possibility of biobanking [21]. In addition, in most cases organoids were derived from relapsed and metastatic tumors but could not be efficiently derived from primary tumors [26, 28].

Interestingly, it has been observed that sarcoma samples exhibit increased stemness and aggressiveness after xenografting in mice [29, 30, 31]. Therefore, deriving organoids from previously established xenografts may represent an alternative strategy to improve the modeling success of primary tumors [12].

Here, we report our experience in deriving BME‐based sarcoma organoids from both patient‐derived samples and murine xenografts. While our attempts to generate patient‐derived organoids (PDOs) encountered challenges consistent with previous reports—namely, low formation efficiency and lack of propagation in subsequent passages—we achieved more promising results using xenografted tissue. Specifically, we established long‐term, biobankable organoid cultures from xenografts derived from both parental and doxorubicin‐resistant (DXR) 143B osteosarcoma cells. Transcriptomic and functional analyses of these models identified the nonreceptor tyrosine kinase SRC as a key mediator of doxorubicin resistance in osteosarcoma.

## 2 Results

### 2.1 Generation of Sarcoma PDOs

To investigate the feasibility of establishing sarcoma organoid cultures using BME matrices, we processed 19 surgically resected tumor samples of patients diagnosed with sarcoma at the Hospital Universitario Central de Asturias (Spain) (Figure 1A and Table S1). Relevant aggregated features of patients are summarized in Figure 1B. Briefly, five female and 14 male patients were included, with adult age being the most frequent. The percentage of bone sarcomas and soft tissue sarcomas assayed was 47 and 53, respectively, chondrosarcoma and osteosarcoma being the most represented tumor types. Most of the tumors (*n* = 15) did not receive any prior treatment before the surgery, while the rest received either systemic chemotherapy (*n* = 1) or radiotherapy (*n* = 2) (Figure 1B). All fresh samples were subjected to mechanical and enzymatic disaggregation protocols and embedded in a Cultrex‐BME matrix as described in *Materials and Methods* and summarized in Table S1. We initially used a M0 culture medium adapted from previously published protocols [32]. With the aim of optimizing M0 medium, we gradually tested several compounds for which pro‐stemness properties have been reported (Table S1). Among all the compounds tested, only IL‐6 and IL‐8, which were reported to promote stemness and tumor growth in both soft tissue sarcomas and bone sarcomas [33, 34], produced slight improvements in the growth of 3D structures from patient‐derived samples. Although these positive effects were based on qualitative observations in a limited number of cases, we opted to retain these factors in subsequent culture media formulations, M1 (M0 + IL‐6) and M2 (M1 + IL‐8) (Table S1).

**FIGURE 1:**
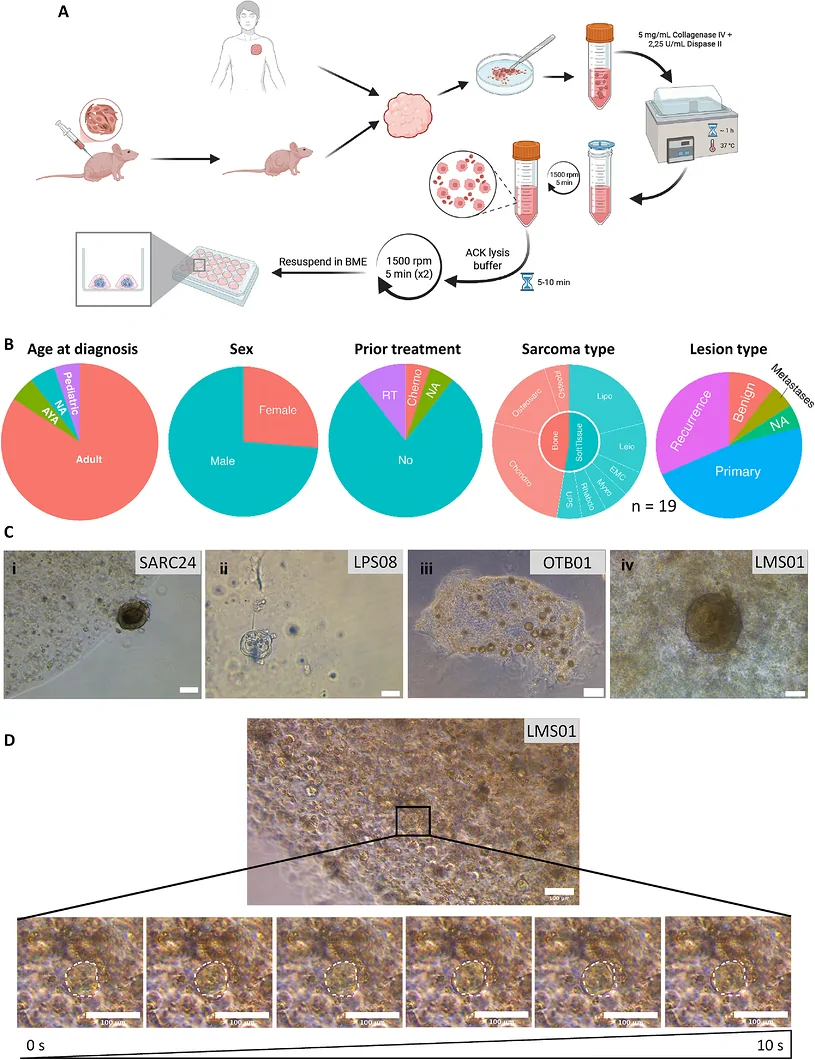
Generation of sarcoma organoids from patient‐derived samples. (A) Scheme showing the process for deriving organoids from patient‐derived samples and tumor xenografts. (B) Distribution of patient's/samples according to age at diagnosis (pediatric: ≤14 years; AYA: 15–39 years; and adult: ≥40 years), sex, type of treatment received prior to surgery (RT: radiotherapy; Chemo: systemic chemotherapy), sarcoma subtype (*Abbreviations*: STS: soft tissue sarcoma; lipo: liposarcoma; Leio: leiomyosarcoma; EMC: extraskeletal myxoid chondrosarcoma; myxo: myxofibrosarcoma; rhabdo: rhabdomyosarcoma; UPS: undifferentiated pleomorphic sarcoma; chondro: chondrosarcoma; osteosarc: osteosarcoma; osteoblast: osteoblastoma) and lesion type. NA: data not available. (C) Representative images of organoids formed from the indicated samples. (D) Spontaneous peristaltic movement observed in the LMS01 sample. Still frames were captured every 2 s from a 10‐s video. The initial outline of the organoid is marked with a white dashed line in each frame. Scale bars: 100 µm.

Using these culture conditions (see Table S1 for specific variations for each case), we observed the growth of 3D structures from seven samples (three liposarcomas, two undifferentiated pleomorphic sarcoma, one leiomyosarcoma, and one osteoblastoma; 37% of the processed samples) (Figure 1C). These structures include large well‐formed organoids displaying an internal dark core and a clear outer ring as in a case of undifferentiated pleomorphic sarcoma (Figure 1Ci), structures resembling cystic organoids in a sample of liposarcoma (Figure 1Cii), or a mix of dense and cystic structures as in a case of osteoblastoma (Figure 1Ciii). Specifically, a case of leiomyosarcoma (LMS‐01) developed a high number of well‐formed dense and cystic organoids (Figure 1C,D) and two of them spontaneously imitated a peristaltic movement that lasted at least 48 h (Figure 1D and Video S1). After 10–12 days of growth, these cultures were disaggregated and reseeded onto new BME matrices. Unfortunately, none of them successfully generated organoids beyond the first passage. This finding highlights the challenges associated with the long‐term maintenance of sarcoma‐derived organoids under the conditions tested.

### 2.2 Generation and Characterization of Organoids From Sarcoma Xenograft Models

Due to the low success rate in the establishment of 3D models directly from patient biopsies, we assayed the derivation of organoids from tumor xenografts generated in immunodeficient mice (Figure 1A). To this end, we grew xenografts from established cell lines, and patient‐derived xenograft (PDX)‐derived cell lines of osteosarcoma (*n* = 7), chondrosarcoma (*n* = 2), Ewing sarcoma (*n* = 1), and undifferentiated pleomorphic sarcoma (*n* = 1) (Table S2) and used freshly harvested samples of these tumors to derive organoids using M2 culture medium. We assayed the derivation of organoids from 14 different tumor xenografts, including subcutaneous (*n* = 11) and intra‐bone (*n* = 3) xenografts models, and we were able to grow 3D organoids from 10 (71.4%) of the samples (Figure 2A). A total of seven of these cultures (50% of the total assayed) could be passaged for at least three passages, subjected to freeze‐thaw cycles and cryopreserved (Table S2). Specifically, two organoid cultures derived from subcutaneous xenografts generated from parental and DXR variants of osteosarcoma 143B cells (143B‐Par and 143B‐DXR, respectively) [35] were able to efficiently grow forming a high number of dense organoids for more than 20 passages (Figure 2B). Histological characterization of 143B‐Par and 143‐DXR organoids confirmed that they recapitulated the main features of the original xenografted tumors, showing compact cell structures with rounded or oval nuclei and focal presence of osteoid‐like material in the cytoplasm of neoplastic cells, causing nuclear displacement. Additionally, xenografts and organoids samples displayed numerous aberrant mitoses (Figure 2C). Moreover, short tandem repeat analysis ratified the origin of these 143B‐derived organoids (Table S3)

**FIGURE 2:**
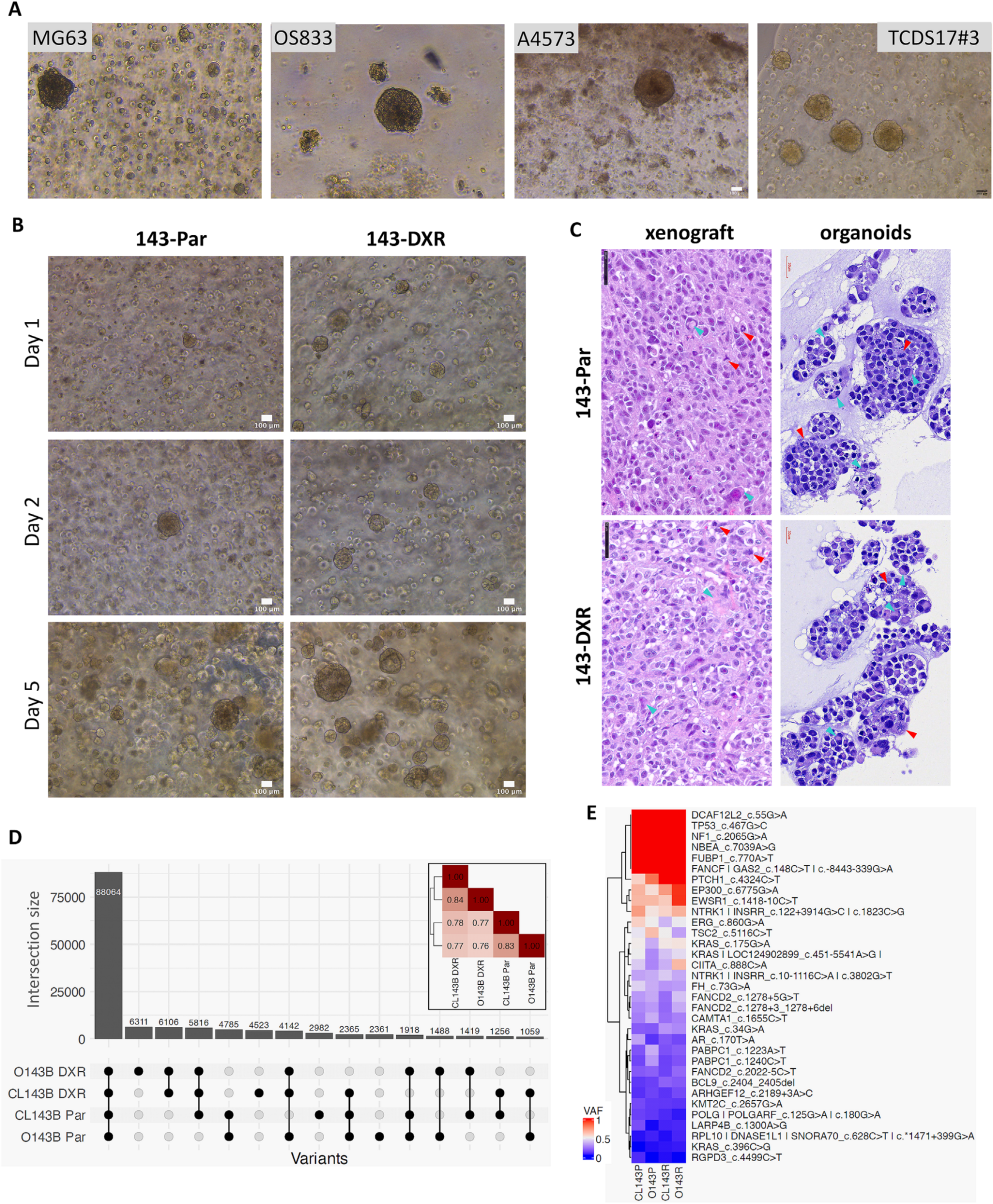
Generation of organoids from sarcoma xenografts. (A) Representative images of organoids formed from tumor xenografts derived out of the indicated cell lines. (B) Organoids derived from the xenografts generated by parental (Par) and doxorubicin‐resistant (DXR) versions of the 143B osteosarcoma cell line. Representative images of the cultures at Days 1, 2, and 5 are shown. (C) Histological analyses (hematoxylin and eosin staining) of xenograft and organoid samples of the indicated models. Aberrant mitoses (red arrowheads) and cells with osteoid‐like material in the cytoplasm (blue arrowheads) are indicated. Scale bars: 100 µm in (A) and (B), 50 µm in (C, left panels) and 30 µm in (C, right panels). (D and E) WES analysis of DNA samples (*n* = 1) from cell line (CL) and organoid (O) cultures of 143B‐Par and 143B‐DXR models. (D) UpSet plot showing the intersections of genomic variants detected by WES in 143B‐Par and 143B‐DXR cell lines (CL) and their corresponding organoid (O) models. The inset shows a correlation plot with Jaccard indices for variant overlap between models. (E) Heatmap showing the VAFs of selected cancer‐related variants across all models.

Whole‐exome sequencing (WES) analysis showed that the 143B‐Par and 143B‐DXR cell lines and their corresponding organoid cultures were genomically similar. After selecting variants with total read counts ≥15 and variant allele frequencies (VAFs) ≥ 0.05, we found that all models shared the majority of the detected mutations, with Jaccard indices for variant overlap of 0.83 for the comparison between 143B‐Par cell lines and organoids and 0.84 for the 143B‐DXR models (Figure 2D). Moreover, comparison between parental and DXR models also revealed a high degree of genomic concordance, with Jaccard indices ranging from 0.75 to 0.80 (Figure 2D). Consistent with this level of similarity, the VAFs of variants shared between cell lines and organoids in both parental and DXR models were highly concordant, with *R* ^2^ correlation values of 0.91 and 0.93, respectively (Figure S1). To evaluate concordance in cancer‐related genes, variants were filtered to retain those with total read counts ≥ 15, VAFs ≥ 0.05, maximum allele frequencies (MAFs) < 0.05, nonsynonymous effects on coding proteins, and affecting genes included in a reported panel of canonical cancer drivers (https://network‐cancer‐genes.org/canonical_drivers.php). This subset of variants also displayed similar VAFs across both parental and DXR models. The most notable difference in VAF values was detected for the PTCH1 c.4324C>T transition, which became homozygous in both resistant models (Figure 2E).

Next, we analyzed the response of 143B‐Par and 143B‐DXR organoids to chemotherapeutic compounds in comparison with that observed in the corresponding 2D monolayer cultures. First, we found that parental organoids were much more resistant to cytotoxic effect of all assayed drugs than the 2D cultures (Figure 3). We also confirmed that 143B‐DXR organoids maintain the DXR phenotype, although, given the increased level of resistance of the parental model, the difference in doxorubicin resistance between 143B‐DXR and 143B‐Par organoids is lower than in the matched 2D models (Figure 3A). Relevantly, as observed in monolayer cultures, 143B‐DXR 3D models were more sensitive to cisplatin (Figure 3B) and methotrexate (Figure 3C). Finally, 3D and 2D models displayed a different response to the antitumor antibiotic mithramycin (MTM), which has previously demonstrated a robust antitumor potential in sarcomas [36], being this compound less effective in the DXR 2D model but slightly more cytotoxic in DXR organoids (Figure 3D).

**FIGURE 3:**
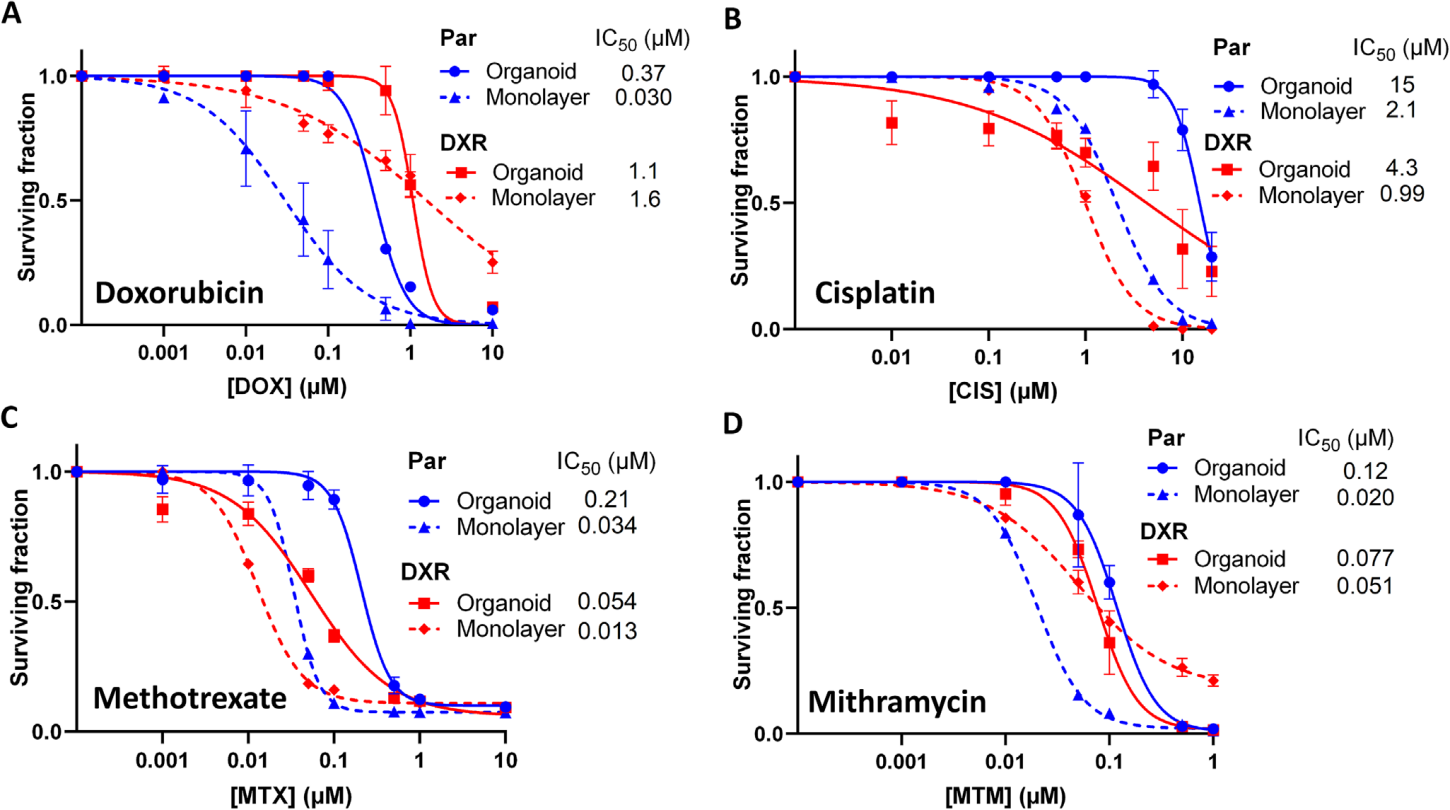
Response of parental and doxorubicin‐resistant organoids to antitumor drugs. Cell viability (CellTiter‐Glo 3D assays) measured after treatment of 143B‐Par (blue) and 143B‐DXR (red) organoids (solid line) and monolayer cultures (dotted line) with increasing concentrations of doxorubicin (DOX) (A), cisplatin (CIS) (B), methotrexate (MTX) (C), and mithramycin (MTM) (D) for 72 h. IC50 values (µM) obtained for each condition are shown. Error bars represent standard deviation of three independent biological replicates.

To further characterize these models, we compared the cell cycle profiles of monolayer and organoid cultures of 143B‐Par and 143B‐DXR cells. The parental and DXR variants in 2D culture, together with parental organoids, showed a similar distribution across all cell cycle phases. In contrast, DXR organoids exhibited a marked accumulation of cells in the G1 phase (Figure S2). These data indicate proliferative heterogeneity between 2D and 3D DXR models, which may contribute to the observed differences in drug response.

Overall, these results demonstrate that sarcoma xenograft samples can be successfully grown as 3D organoids that retain the main histological and molecular features of the original specimens and can be used as models for drug testing.

### 2.3 Gene Expression Profiling of Parental and DXR Osteosarcoma Organoids

To unravel new molecular traits with potential roles in promoting drug resistance in osteosarcoma, we performed RNA‐seq analyses of 143B‐Par and 143B‐DXR organoids. Comparison between the transcriptomics profiles of both models revealed an intense level of gene expression modulation (Figure 4A) with similar number of genes being upregulated (*n* = 810) and downregulated (*n* = 895) in 143B‐DXR organoids (Figure 4B). Gene set enrichment analysis revealed increased transmembrane transporter activity and downregulation of pathways related to the regulation of the inflammatory response and integrin binding, among other molecular and functional processes (Figure S3).

**FIGURE 4:**
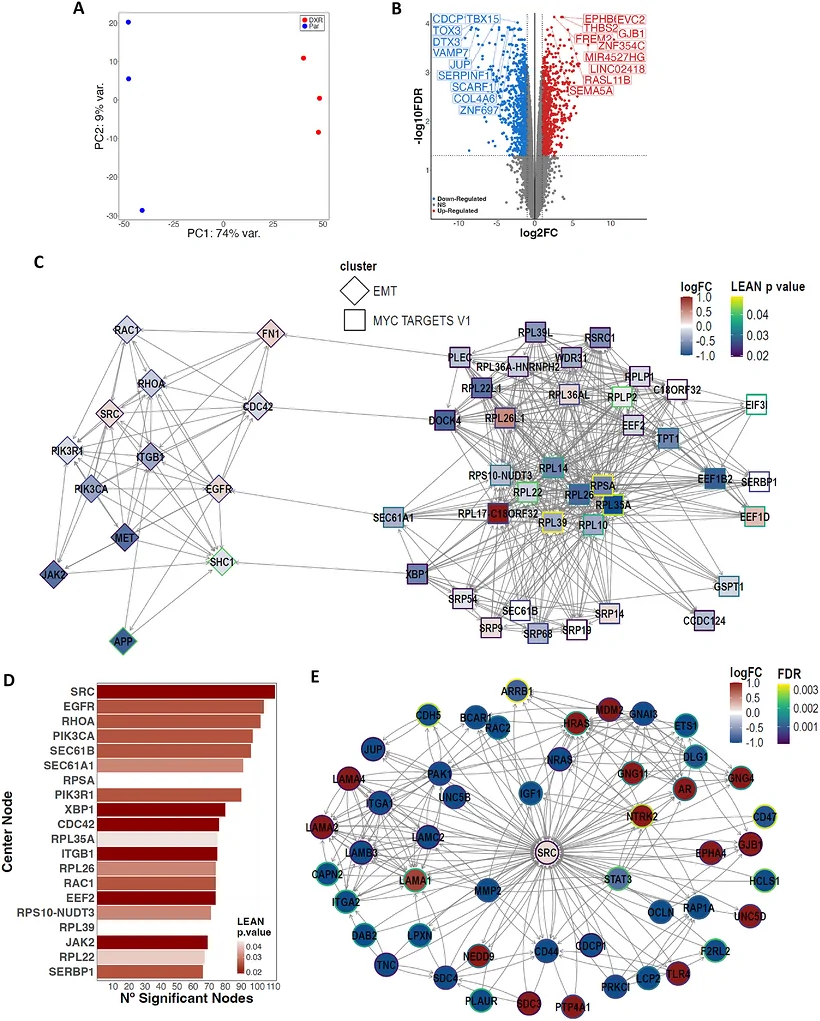
Transcriptomic characterization of the resistant phenotype in 143B‐DXR organoids. (A) Principal component analysis (PCA) of samples from three biological replicates of 143B‐Par (Par) and 143B‐DXR (DXR) organoids according to the top 500 most variable genes between conditions. (B) Volcano plot representing genes significantly upregulated (red) and downregulated (blue). A gene is considered differentially expressed if it has an FDR < 0.05 and a |log2FC| ≥ 1. The 10 most upregulated and downregulated genes are indicated. (C) Network representation of the statistical significance of the aggregate observed transcriptomic modulation of the gene and its interactors after applying LEAN. Only central nodes whose sub‐networks are composed of 50 nodes or more are represented. A scheme of the network including all significant nodes is shown in Figure S4A. The color of the node indicates the log2FC from the differential expression analysis, while the color of the node border indicates corrected *p* value of the aggregate transcriptomic modulation according to LEAN. An unsupervised clustering of this network was performed, and the overrepresented pathway in each group is represented by the node shape. (D) Bar plot showing the top 20 hub nodes whose local networks contain a higher number of significant nodes according to LEAN. The bar color indicates the significance level for that node according to LEAN. (E) Representation of the local network centered on SRC, where node color indicates the log2FC and the border color represents the FDR from the differential expression analysis. Only the 50 most significant nodes are represented. A scheme of the SRC sub‐network including all significant nodes is shown in Figure S4B.

We used the Local Enrichment Analysis (LEAN) method to identify centers of local gene networks with statistically significant aggregate transcriptomic modulation (false discovery rate [FDR] < 0.05) [37]. Figure 4C shows the resulting two networks of interrelated central signaling nodes whose sub‐networks are composed of 50 nodes or more: one comprising a set of c‐Myc targets, and another related to epithelial‐mesenchymal transition. When all significant central nodes are considered, two additional sub‐networks related to NFκB signaling and glycolysis factors emerge (Figure S4A). Among the filtered nodes, the nonreceptor protein tyrosine kinase SRC is the most significantly altered node and the one with the most extensive subnetwork (Figures 4D,E and S4B). Of note, other relevantly altered nodes, such as those controlled by EGFR3, RAC1, RHOA, PI3K, and so on, are mechanistically related with SRC‐mediated signaling [38] (Figure 4D). Relevantly, deregulated SRC signaling has been proved to play relevant roles in tumor proliferation, migration, invasion and dissemination [39, 40]. Likewise, the role of SRC in mediating resistance to different antitumor treatments has also been reported [41, 42, 43].

Overall, these results suggest that SRC plays a key role in doxorubicin resistance in osteosarcoma.

### 2.4 The Inhibition of SRC Signaling Sensitizes Drug‐Resistant Osteosarcoma Organoids to Doxorubicin

Given the relevant roles that SRC signaling may play in tumor progression and drug resistance, we assayed whether the inhibition of SRC may increase the antitumor activity of doxorubicin in our osteosarcoma organoids. First, we treated 143B‐Par and 143B‐DXR organoids with two different concentrations of doxorubicin (0.1 and 1 µM) alone or in combination with the SRC inhibitor dasatinib. We found that a concentration of 1 µM of this inhibitor did not induce any significant cytotoxic effect in any of those models. When comparing the effects of the treatments with doxorubicin alone and the combo, we observed a sensitizing effect of dasatinib in 143B‐Par organoids when this inhibitor was combined with the lowest concentration of doxorubicin. Likewise, we found a stronger effect of the combination in 143B‐DXR organoids when using the higher concentration of doxorubicin (Figures 5A and S5A). In this case, the response of the resistant model to the combination recapitulated that of the parental model to doxorubicin monotherapy, indicating a potent chemosensitizing effect following SRC inhibition. Consistently, this effect was further confirmed using another SRC inhibitor, saracatinib, which also significantly reduced viability in 143B‐DXR cells when combined with both tested concentrations of doxorubicin (Figures 5B and S5B).

**FIGURE 5:**
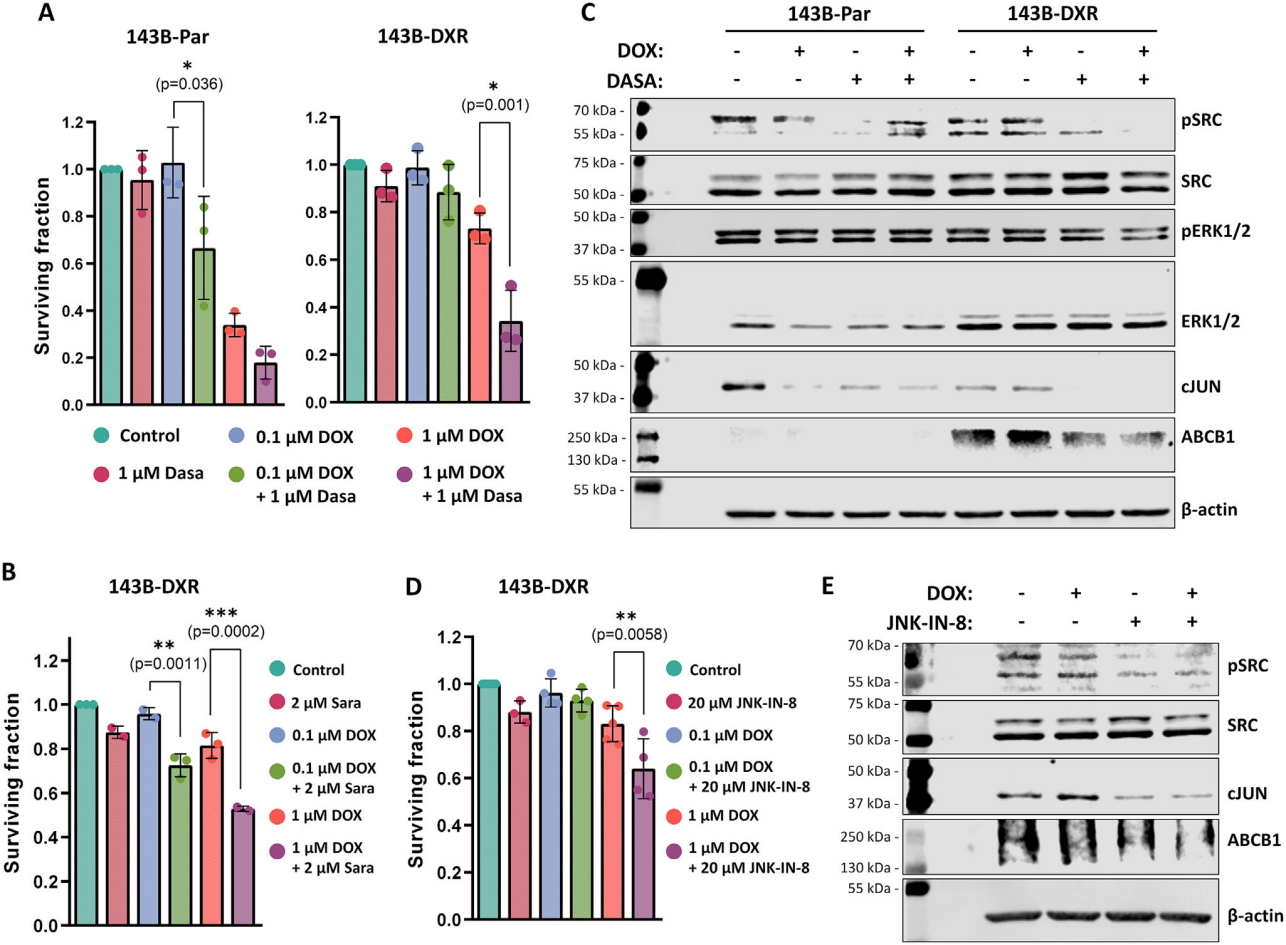
SRC inhibition improves the response to doxorubicin in 143B‐DXR organoids through the suppression of cJUN–mediated ABCB1 expression. (A and B) Cell viability (CellTiterGlo‐3D assays) measured after treatment of 143B‐Par and/or 143B‐DXR organoids with DMSO (control) and the indicated concentrations of doxorubicin (DOX) alone or in combination with SRC inhibitors dasatinib (Dasa) (A) or saracatinib (Sara) (B) for 72 h. (C) Western blotting analyses of the indicated proteins in control conditions or after the treatment of 143B‐Par and 143B‐DXR organoids with 1 µM doxorubicin, 1 µM dasatinib, or the combination of both for 24 h. (D) Cell viability measured after treatment of 143B‐DXR organoids with DMSO (control) and the indicated concentrations of doxorubicin (DOX) alone or in combination with the JNK–IN‐8 for 72 h. (E) Western blotting analyses of the indicated proteins in control conditions or after the treatment of 143B‐DXR organoids with 1 µM Doxorubicin, 20 µM JNK–IN‐8, or the combination of both for 24 h. Error bars in panels A, B, and D represent standard deviation of three independent biological replicates and asterisks indicate statistically significant differences between doxorubicin monotherapy and combination treatments based on one‐way ANOVA tests. *: *p* < 0.05; **: *p* < 0.01; ***: *p* < 0.001. Other significant differences between the treatments shown in panels A, B, and D are depicted in Figure S5. Quantification of Western blotting analyses (*n* = 1) are shown in Figures S7 and S8.

To assess whether the efficacy of this combinatory treatment is also observed in other DXR models, we treated three available DXR osteosarcoma 2D cell lines (143B‐DXR, SaOS‐2‐DXR, and MG‐63‐DXR). We found that, consistent with the effect observed in 143B organoids, dasatinib significantly sensitized all DXR models to doxorubicin, supporting the role of SRC as a common vulnerability in DXR osteosarcoma models (Figure S6).

The activation of SRC is usually mediated by its association with focal adhesion kinase, which induced SRC autophosphorylation at Y419, leading to its activation [44]. Downstream, activated SRC may promote the activation of prosurvival signals like those mediated by PI3K–AKT, ERK, or JNK [39, 42]. To gain insight about the molecular basis of the sensitizing effect of dasatinib, we analyzed the activation of SRC, ERK, and cJUN in 143B‐Par and 143B‐DXR organoids following the treatment with doxorubicin alone or in combination with the SRC inhibitor. Western blotting analyses showed that a 24‐h treatment with 1 µM dasatinib efficiently inhibited SRC (Y419) phosphorylation in both organoid models while doxorubicin alone did not induce significant changes in SRC phosphorylation levels. Notably, when combined with doxorubicin, dasatinib could only partially reduce SRC phosphorylation in 143B‐Par cells, whereas it completely prevented the activation of SRC in 143B‐DXR cells (Figures 5C and S7). Downstream SRC activation, we observed that both doxorubicin and dasatinib alone partially inhibited ERK phosphorylation in 143B‐Par organoids. On the other hand, the 143B‐DXR model presented higher levels of phosphorylated ERK isoforms, and doxorubicin and/or dasatinib failed to reduce them. Similarly, treatment with doxorubicin and dasatinib alone reduced cJUN levels in 143B‐Par cells with no additional inhibitory effect observed when combined. Relevantly, in 143B‐DXR organoids, while doxorubicin failed to reduce cJUN levels, dasatinib alone or in combination with doxorubicin efficiently inhibit cJUN activation. Finally, as previously shown, 143B‐DXR organoids highly overexpress ABCB1, an ATP‐dependent drug efflux pump for xenobiotic compounds, and both dasatinib monotherapy and the combination treatment significantly reduce its expression (Figures 5C and S7).

To confirm the mechanistic role of cJUN repression following SRC inhibition, we treated 143B‐DXR cells with the selective JNK inhibitor JNK–IN‐8, which prevents JNK‐mediated phosphorylation of cJUN, thereby reducing its stability and transcriptional activity [45]. Similar to SRC inhibitors, this compound significantly enhanced the cytotoxic effect of 1 µM doxorubicin (Figures 5D and S5C). Also consistent with the effects observed upon SRC inhibition, JNK–IN‐8‐mediated reduction of cJUN levels led to decreased ABCB1 expression, particularly in cells treated with the combination of doxorubicin and the JNK inhibitor (Figures 5E and S8).

These results suggest that the chemosensitizing effect of dasatinib observed in the DXR models is mediated through its ability to inhibit cJUN‐mediated ABCB1 expression, thereby facilitating intracellular accumulation of doxorubicin.

### 2.5 Inhibition of SRC Improves the Response of Drug‐Resistant Osteosarcoma Xenografts to Doxorubicin in Vivo

We next assessed whether the chemosensitizing effect of SRC inhibition is also observed in vivo. To this end, we established xenografts in mice using 143B‐DXR organoids and treated them with vehicle (control), doxorubicin, dasatinib, or the combination. As expected, DXR tumors did not respond to either doxorubicin or dasatinib alone. However, the combination treatment significantly inhibited tumor growth (Figure 6A) without causing weight loss (Figure 6B) or other adverse effects.

**FIGURE 6:**
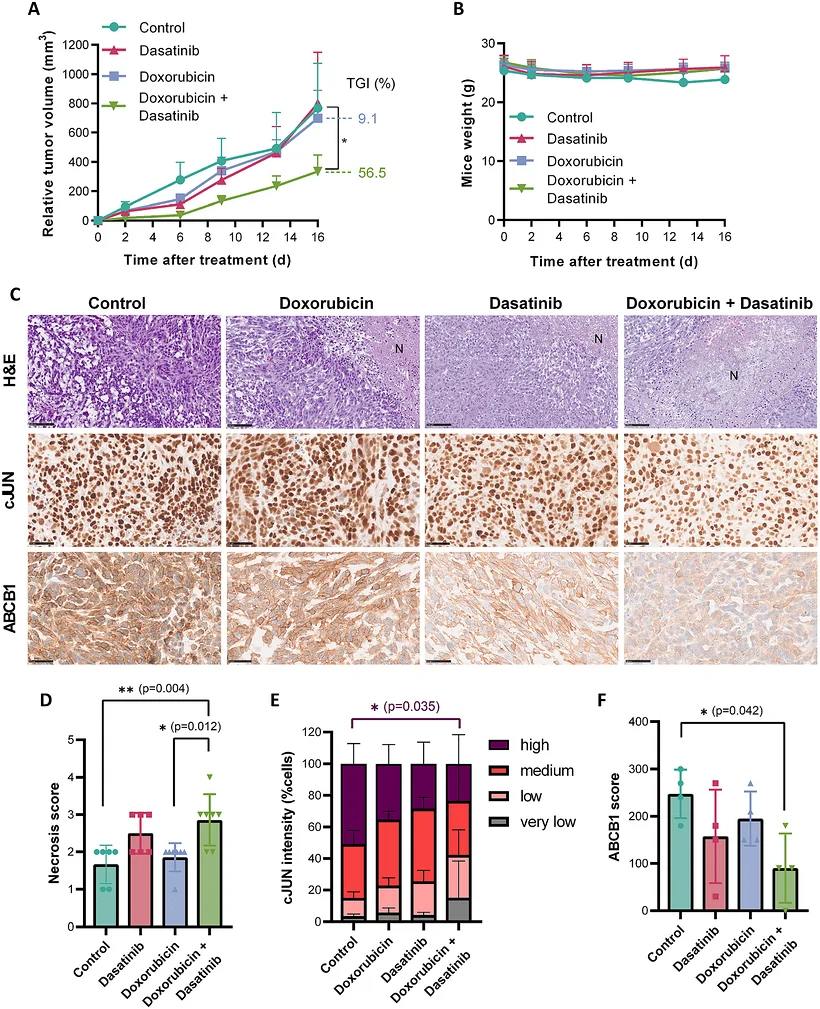
In vivo antitumor effect of the doxorubicin–dasatinib combination. Xenografted mice generated from 143B‐DXR organoids were randomly assigned to the indicated treatment groups (*n* = 7). (A) Curves representing the mean relative tumor volume of xenografts during treatment. Drug efficacy, expressed as the percentage of tumor growth inhibition (TGI), is indicated. (B) Changes in body weight during treatment. (C–F) Histological analysis of tumors collected at the end of treatment. (C) Representative images of hematoxylin and eosin (H&E) staining (top panels) and immunostaining for cJUN (middle panels) and ABCB1 (bottom panels). Necrotic areas (N) are indicated. Scale bars: 100 µm for H&E and 50 µm for cJUN and ABCB1. (D) Mean necrotic score. (E) Quantification of the percentage of cells showing different levels of cJUN staining intensity. (F) Mean ABCB1 score. Error bars represent SEM in panels A and B and SD in panels D–F. In panels A and B, data are from all mice (*n* = 7 per group); in panel D, from tumors of all mice reaching the experimental endpoint (*n* = 6 for control and dasatinib groups; *n* = 7 for doxorubicin and combination groups); and in panels E and F, from four randomly selected tumors per group. Asterisks indicate statistically significant differences between groups (*p < 0.05; **p < 0.01). In panel E, the asterisk indicates significance for the high‐intensity group. Statistical analyses were performed using one‐way ANOVA followed by Tukey's multiple‐comparison test (D and F) or two‐way ANOVA followed by Tukey's multiple‐comparison test (A and E).

Histological analysis showed that tumors treated with the doxorubicin–dasatinib combination exhibited significantly larger necrotic areas than control and doxorubicin‐treated tumors (Figure 6C,D). Consistently, tumors receiving the combination treatment showed significantly reduced expression of cJUN (Figure 6C,E) and ABCB1 (Figure 6C,F) compared with control samples.

These findings validate in vivo the ability of dasatinib to re‐sensitize DXR osteosarcoma cells.

## 3 Discussion

The generation of BME‐based organoids from sarcomas is not as well developed as from epithelial cancers. Although notable progress has been made in recent years, the initial requirements established for organoid models—namely, three‐dimensional, tissue‐derived cultures with self‐renewal and differentiation potential that can be expanded long‐term in serum‐free conditions mimicking the in vivo stem cell niche [7]—have not been achieved in most reported sarcoma organoid models. In this work we encountered similar challenges to those described in previous reports when attempting to establish BME‐based organoids from patient‐derived samples. For example, we observed the growth of scattered organoid‐like structures in only seven out of 19 (37%) processed samples. Notably, in one case of leiomyosarcoma, several 3D structures displayed functional peristaltic movements. Nonetheless, none of the cultures could be successfully passaged or cryopreserved. These findings are in stark contrast to the high success rates reported by Nie et al., who achieved long‐term BME‐based organoid cultures from 90 to 95% of osteosarcoma biopsies (*n* = 20) and surgical specimens (*n* = 12) [46]. A key difference that may help explain these discrepancies is that, unlike our study, Nie et al. included 10% fetal bovine serum (FBS) in their culture medium.

Another factor that may influence the efficiency of organoid derivation from sarcomas is the type of lesion being modeled. For instance, Savary et al. assayed the derivation of scaffold‐free organoids from fusion‐negative rhadomyosarcomas [26]. Among 11 processed samples, optimal 3D growth was achieved only in five samples derived from recurrent/metastatic aggressive cases, but not in any of the six primary samples analyzed, which instead could be expanded as 2D cultures. Similarly, Meister et al. established 19 rhabdomyosarcoma models from both primary and relapsed tumors, but all failed to grow as 3D cultures and were maintained as 2D monolayers [23].

These observations suggest that primary and/or less aggressive sarcomas may lack the intrinsic capacity to grow as 3D organoids in BME‐supported, serum‐free conditions. However, during progression to more advanced or aggressive stages, sarcoma cells may acquire molecular traits that support a 3D growth phenotype when cultured ex vivo. In this context, other organoid culture systems [10, 11, 12], such as scaffold free cultures [18, 26], biomimetic scaffolds that support tumor growth and mimic specific microenvironment [22, 47, 48, 49], and/or the use of alternative culture methods like the air–liquid interface technique or self‐assembly protocols [25, 50, 51, 52] have shown promise in modeling patient‐derived sarcomas and may help overcome the limitations associated with BME‐based 3D cultures.

It has been reported that tumor xenografting in mice can increase the aggressiveness of cancer cells [29, 30, 31], and therefore many works have generated organoids from previously established tumor xenografts as a way to increase the success rate of organoid formation [53, 54]. Using this approach, we successfully established organoids from xenograft tissue derived from various bone sarcoma cell lines. The xenograft tissues used in these experiments were generated in moderately immunodeficient athymic nude mice. Therefore, further optimization of this strategy may lie in the use of highly immunodeficient mouse strains [55], which could facilitate xenograft engraftment and growth, thereby potentially increasing the likelihood of successful downstream organoid derivation.

In particular, we generated and characterized a novel DXR osteosarcoma organoid model along with its parental counterpart, both derived from xenografts formed by 143B cells. These 143B‐derived models were propagated long term as dense cultures of well‐formed 3D organoids and were amenable to biobanking. Furthermore, these organoids constitute a unique resistant/parental paired system for studying drug resistance in osteosarcoma. We used LEAN network analysis [37] to robustly identify the most significant centers of transcriptomic change between the parental and DXR 143B organoids. This analysis identified a signaling node controlled by SRC as a highly connected center of the differential transcriptome in DXR organoids. Confirming the potential role of this pathway in the mediation of the resistant phenotype in osteosarcoma, we found that the inhibition of this pathway using dasatinib or saracatinib was able to significantly enhance the cytotoxic activity of doxorubicin in the 143B‐DXR organoids both in vitro and in vivo. In line with these findings, a drug screen in a panel of patient‐derived sarcoma cell cultures identified dasatinib as an active compound in tumors harboring chromosomal translocations [56]. Also supporting a role for SRC in drug resistance in sarcomas, it has been reported that the SRC inhibitor A‐770041 was able to reverse paclitaxel and doxorubicin resistance in osteosarcoma cell lines [41].

Further analysis of SRC downstream signaling revealed that the inability of doxorubicin to inhibit ERK activation and cJUN expression in resistant organoids may underlie the observed resistance phenotype. Although dasatinib treatment did not affect ERK1/2 activation in the resistant model, our in vitro and in vivo findings indicate that the chemosensitizing effect of this SRC inhibitor is mediated via suppression of the SRC/cJUN/ABCB1 axis. It is plausible to hypothesize that dasatinib‐induced downregulation of ABCB1 may enhance the efficacy of doxorubicin in chemoresistant organoids. Describing a similar mechanism of chemo‐sensitization, a recent study reported that resistance to covalent inhibitors of KRAS–G12C in non‐small cell lung cancer is driven by cJUN‐mediated induction of ABCC1 expression. Moreover, this study showed that dasatinib enhances the efficacy of KRAS–G12C‐targeted therapies by inhibiting SRC‐dependent cJUN activation [43].

In this study, we highlighted the challenges associated with deriving BME‐based organoids from patient‐derived sarcoma samples. As an alternative approach, we demonstrated that 3D organoids with long‐term growth potential can be successfully generated from sarcoma xenografts. A direct comparison of the ability of the same patient‐derived samples to generate organoids, either directly or following xenograft expansion, will provide definitive insight into whether this intermediate step improves the success rate of sarcoma organoid derivation. Nevertheless, our results suggest that xenograft growth represents a useful strategy to enhance organoid establishment.

It is also important to note that some organoid models generated in this study were developed from established cell lines xenografted in mice, rather than from patient‐derived samples, and therefore lack immediate clinical applicability. Nonetheless, these models represent a significant addition to the repertoire of available 3D sarcoma models. Specifically, the paired 143B‐Par and 143B‐DXR organoids constitute a novel 3D platform for investigating drug resistance in osteosarcoma. In this context, we showed that inhibition of SRC‐mediated signaling with dasatinib can reverse doxorubicin resistance in osteosarcoma by suppressing cJUN‐mediated ABCB1 expression. Future efforts aimed at expanding the panel of DXR osteosarcoma organoids, also including patient‐derived models, may enable further validation of this and other potential doxorubicin‐sensitizing therapies, as well as the identification of biomarkers associated with treatment response.

## 4 Materials and Methods

### 4.1 Establishment of Sarcoma Organoids

The protocol to establish sarcoma organoids were adapted from previously described protocols for the generation of organoids from epithelial tumors [32, 57, 58]. First, the tumor was mechanically fragmented using scalpels and these fragments were enzymatically digested to a single cell suspension by incubating them for 1 h at 37°C with the digestion medium, mixing every 10–15 min by shaking vigorously and by pipetting up and down. Digestion medium consists of 5 mg/mL Collagenase IV (Sigma–Aldrich, San Louis, MO, USA), 2.25 U/mL Dispase II (Roche, Mannheim, Germany), 10 µM ROCK inhibitor Y‐27632 (Biogems, Westlake Village, CA, USA), and 100 µg/mL primocin (InvivoGen, San Diego, CA, USA), dissolved in basal medium (BM), which is advanced Dulbecco's modified Eagle medium/F‐12 (Gibco, Carlsbad, CA, USA) supplemented with 1% (v/v) penicillin–streptomycin (10,000 U/mL; Gibco), 1% (v/v) GlutaMAX (Gibco), and 1% (v/v) HEPES buffer solution (1 M; Gibco). After 1 h, the digestion process was stopped by dilution with BM and the remaining tissue fragments were filtered out using a 100 µm cell strainer. The cell suspension was then centrifuged at 1500 rpm for 5 min at 4°C and the pellet was resuspended in ACK lysing buffer (Gibco) and incubated for 5–10 min at RT to remove erythrocytes from the sample. After a new centrifugation, the pellet was resuspended in BME (Cultrex Basement Membrane Extract; Bio‐Techne, Minneapolis, MN, USA) and plated in preheated 24 well suspension plates as drops of 10 µL each. Afterward, the plate was placed into a humidified incubator at 37°C for 20–30 min to let the BME solidify following the hanging drop method. Finally, the plate was flipped, and the organoids were cultured in growth medium at 37°C in a humidified atmosphere (5% CO_2_ and 95% air), changing the medium every 2–3 days and splitting them each 7–10 days. The growth medium is composed by BM supplemented with 10 mM nicotinamide (Sigma–Aldrich), 1.25 mM N‐acetyl‐l‐cysteine (Sigma–Aldrich), 2% (v/v) B27 supplement (minus vitamin A; Gibco), 0.05 µg/mL EGF (PeproTech, London, UK), 0.005 µg/mL FGF‐2 (PeproTech), 1 µM Forskolin (Bio‐Techne), 0.1 µg/mL R‐spondin‐1 (PeproTech), 3 µM CHIR99021 (Sigma–Aldrich), 0.01 µg/mL FGF‐10 (PeproTech) (M0 medium). This M0 medium was then optimized by adding 20 ng/mL IL‐6 (PeproTech) (M1 medium) and 20 ng/mL IL‐8 (PeproTech) (M2 medium). Other compounds tested during the optimization process and not maintained in subsequent optimized versions of the medium were A83‐01 (0.5 µM; Bio‐Techne), BMP2 (2 ng/mL; PeproTech), PDGF‐α (10 ng/mL; PeproTech), PDGF‐β (10 ng/mL; PeproTech), TGF‐β (10 ng/mL; PeproTech), XMU‐MP‐1 (10 ng/mL; MedChemExpress, Monmouth Junction, NJ, USA), and Pam3Cys (20 ng/mL; InvivoGen). Besides, for the first two passages after the establishment of the organoids, the different versions of the growth medium were supplemented with 10 µM ROCK inhibitor Y‐27632 and 100 µg/mL primocin.

To split the organoids, first the BME domes were digested by incubation for 30 min–1 h with 1 mg/mL Dispase II (Roche) at 37°C in a humidified incubator. Then, the digestion process was stopped by dilution with cold BM and the matrix‐free organoid solution was centrifuged at 1500 rpm for 5 min at 4°C. The pellet was resuspended in BM and the organoids were manually disaggregated by pipetting vigorously up and down with a p10 tip and centrifuged again at 1500 rpm for 5 min at 4°C. Finally, the single‐cell pellet was resuspended in the appropriated BME amount, depending on the split ratio (usually between 1:2 and 1:4), and processed and cultured as described above.

### 4.2 Generation of Tumor Xenografts as Starting Tissue Material for Organoid Culture

Human osteosarcoma cell lines 143B (CRL‐8303), SaOS‐2 (HTB‐85), and MG‐63 (CRL‐1427) were obtained from ATCC (Manassas, VA, USA). Human Ewing sarcoma cell line A4573 was obtained from the EuroBoNet consortium. DXR variant of 143B cell line was developed by continuous stepwise exposure as previously described [35]. Patient‐derived chondrosarcoma models T‐CDS‐17#3 and T‐CDS‐17#4 were established as previously described [31]. PDX osteosarcoma cell lines OS833, OS742, and OS186 were kindly donated by Sweet‐Cordero Lab (UCSF, San Francisco, CA, USA) [55]. All cell lines were cultured in Dulbecco's modified Eagle medium (Corning, AZ, USA) supplemented with 10% (v/v) FBS (Gibco), 1% (v/v) penicillin–streptomycin (10,000 U/mL; Gibco), and 1% (v/v) GlutaMAX (Gibco) at 37°C in a humidified atmosphere (5% CO_2_ and 95% air). All cultures were regularly to discard mycoplasma contamination using the Biotools Mycoplasma Gel Detection kit (B&M LABS, Madrid, Spain). The identity of cell lines has been authenticated by Short Tandem Repeats analysis.

These cell lines were inoculated in female 6 weeks‐old female athymic nude‐Foxn1nu mice (Envigo, Barcelona, Spain) subcutaneously or intra‐bone as previously described [31, 35]. Once the tumor volumes were in an optimal size, mice were sacrificed by cervical dislocation and tumors were extracted and processed for organoid culture as described above.

### 4.3 Histological Analyses

Organoids were fixed inside the BME domes in 4% formaldehyde and 0.1% glutaraldehyde for 30 min at RT and embedded in Histogel (Epredia, Portsmouth, NH, USA) to perform histological analyses as previously described [6]. Xenograft tumor samples were also fixed in 4% formaldehyde and embedded in paraffin for immunohistochemistry analyses using the following primary antibodies: anti‐MDR1/ABCB1 (E1Y7S, #13978) from Cell Signaling (Danvers, MA, USA) and anti‐cJUN (24909‐1) from Proteintech (Rosemont, IL, USA). Slides were scanned using the NanoZoomer‐SQ digital scanner (Model C13140; Hamamatsu Photonics, Hamamatsu City, Japan). Histopathological evaluation was performed by two histopathologists. Necrotic scores were assigned according to the extent of the necrotic area (1, 0–25%; 2, 25–50%; 3, 50–75%; 4, >75%). ABCB1 expression was quantified using a semiquantitative scoring system based on the percentage of stained cells and staining intensity (0, no expression; 3, high intensity). The final ABCB1 score for each sample was calculated as the product of both parameters. Nuclear cJUN staining was automatically quantified using QuPath v0.7.0 software. At least 7000 cells from two different fields were classified into four intensity groups (1: 0–0.3; 2: 0.3–0.6; 3: 0.6–0.9; and 4: >0.9 optical density arbitrary units) and the percentage of cells in each group was determined.

### 4.4 Drugs

Doxorubicin, methotrexate, cisplatin, dasatinib, and saracatinib were purchased from Selleckchem (Houston, TX, USA) and JNK–IN‐8 from MedChem. MTM was synthesized by EntreChem SL (Oviedo, Spain) [36]. Stocks were prepared as 10 mM solutions in sterile DMSO or dimethylformamide in the case of cisplatin.

### 4.5 Whole‐Exome Sequencing

Total DNA samples were extracted from cultures of 143B‐Par and 143B‐DXR in 2D and organoid models using QIAmp DNA Mini Kit (Qiagen, Valencia, CA, USA) following the manufacturer's instructions. Paired‐end libraries were prepared using the SureSelect Human All Exon V8 kit (Agilent Technologies, Santa Clara, CA, USA) and sequencing on an Illumina platform. All sequencing data files were evaluated for quality control using fastQC and adapters and poor‐quality paired‐end reads were removed using fastp. These preprocessed reads were aligned to the GRCh38 human genome using BWA‐mem, sorted with SAMtools, and optical and PCR duplicates were removed using Sambamba. The average nucleotide coverage was approximately 170×. Single nucleotide variants (SNVs) and small insertions/deletions (indels) were identified using a variant‐calling algorithm developed by Dreamgenics (Oviedo, Spain). For each variant identified in a given sample, supporting information at the corresponding genomic position was subsequently retrieved across all remaining samples, irrespective of whether the variant had been independently called in those samples. Variants were annotated using information from functional databases (RefSeq and PFAM), population frequency resources (gnomAD, dbSNP, 1000 Genomes Project, ESP6500, ExAC), in silico functional prediction tools (dbNSFP and dbscSNV), and clinical annotation resources (ClinVar and Human Phenotype Ontology).

Downstream analyses were performed using R statistical software (v4.4.1) and Bioconductor packages (v3.19). A called variant was considered present if it met the following criteria in at least one sample: sequencing depth ≥ 15 reads and VAF ≥ 0.05. For the selection of cancer‐related genes, additional filtering criteria were applied, including: MAF < 0.05; predefined variant protein effects (“FIRST_MET,” “STOP_GAINED,” “STOP_LOST,” “CANONICAL_SPLICING,” “FRAMESHIFT,” “NO_SYN,” “SPLICING,” “INFRAME”); and inclusion of the affected gene in the canonical cancer driver list from the Network of Cancer Genes & Healthy Drivers (NCG7.2). The Jaccard similarity index was used to assess the overlap between mutational profiles across samples. In addition, Pearson's correlation coefficient and *R* ^2^ values were calculated using the VAFs of variants shared between models.

### 4.6 RNA Sequencing and Data Analysis

Total RNA samples were extracted from triplicated cultures of 143B‐Par and 143B‐DXR organoid models using GeneMatrix Universal RNA Purification Kit (EURX, Gdańsk, Poland). cDNA libraries were prepared by polyA enrichment and checked for quality and quantified using an Agilent 2100 Bioanalyzer (Agilent Technologies). Paired‐end sequencing was performed on an Illumina Novaseq X Plus Series (Illumina, San Diego, CA, USA) using 150‐base reads (6 Gb per sample). All sequencing data files were evaluated for quality control and adapters and poor‐quality paired‐end reads were removed using fastp (v.0.23.4) and these preprocessed reads were pseudo‐aligned to the GRCh38 human genome and quantified at the transcript‐level using the mapping‐based mode of the Salmon package (v.1.10.3).

Analyses of gene expression data were performed using packages from R statistical software (v.4.4.1) and Bioconductor (v.3.19). Transcript‐level information was summarized at the gene‐level for both exploratory and differential analyses using the tximeta package (v.1.22.1), normalizing counts as transcripts per million (TPM) with the lengthScaledTPM option. Nonexpressed genes were discarded using filterByExpr algorithm from edgeR package (v.4.2.1). Differential gene expression analysis was performed by applying empirical Bayes moderated t‐statistics on gene‐wise linear models using limma (v.3.60.6). After adjusting *p* values for multiple testing using the Benjamini–Hochberg method, differentially expressed genes were defined as those with FDR < 0.05 and |log2 (FC)| > 1 (FDR, FC: fold change). Pathway enrichment analysis between both conditions were performed using the clusterProfiler package (v.4.12.6) on the gene sets from the Molecular Signatures Database (MSigDB) Reactome and Gene Ontology collections, accessed via the msigdbr package (v.7.5.1). Gene networks were identified that displayed significant aggregate transcriptomic modulation between different transcriptomes. LEAN was selected for this purpose over alternative analytical network‐based approaches due to its suitability to leverage existing knowledge of gene–gene interactions, and its capacity to provide true estimates of statistical significance of observed transcriptomic differences [37].

### 4.7 Cell Viability Assays

Cell viability of organoids after the treatment with increasing concentrations of different drugs was assayed using the CellTiter‐Glo 3D Cell Viability Assay (Promega, Madison, WI, USA) after 72 h‐treatments. Briefly, 2 days preformed organoids were transferred to a white opaque 96 well plate and cultured with the treatments for 72 h. Then 1:1 (v/v) of CellTiter‐Glo 3D Cell Viability Assay were added and incubated for 30 min at RT, shaking vigorously during the first 5 min. Finally, the viability was measured as luminescence values. Cell viability in monolayer cultures was evaluated using the cell proliferation reagent WST‐1 (Roche) after 72 h‐treatments as previously described [6]. IC_50_ for each treatment was determined by nonlinear regression using GraphPad Prism 10.2.3 software (Graphpad Software Inc, CA, USA).

### 4.8 Cell Cycle Analysis

Cells and organoids were seeded at high density and allowed to grow 24 h. Following this time, they were collected, fixed with 1 mL of ice‐cold 70% ethanol added dropwise while gently vortexing, and stored at −20°C for at least 24 h. Prior to staining, fixed cells/organoids were washed twice with PBS, and the cell/organoid pellet was then resuspended in 500 µL of FxCycle PI/RNAse staining solution (Invitrogen, CA, USA) and mixed thoroughly. Samples were incubated for 15 min at RT in the dark, filtered, and analyzed by flow cytometry using a Cytek Northern Lights (NL)‐CLC spectral flow cytometer (Cytek Biosciences, Amsterdam, The Netherlands). Cell cycle distribution was determined using the FlowJo v10.8 software (BD Biosciences, San Diego, California, USA).

### 4.9 Western Blotting

Whole cell protein extraction and Western blot analysis were performed as previously described [35]. Primary antibodies used in these analyses were as follow: anti‐ABCB1 ([13342], 1:1000 dilution) and anti‐ERK1/2 ([9102], 1:1000 dilution) from Cell Signaling; anti‐phospho‐ERK1 (T202/Y204)/ERK2 (T185/Y187) ([MAB1825], 1000 dilution), anti‐cJUN ([MAB2670], 1:500 dilution), and anti‐phospho‐SRC (Y419) ([AF2685], 1:200 dilution) from Bio‐Techne; anti‐SRC ([sc‐8056], 1:500 dilution) from Santa Cruz Biotechnology (Dallas, TX, USA); and anti‐ß‐Actin ([A5441], 1:5000 dilution) from Sigma–Aldrich. IRDye 800CW and IRDye‐680RD from LI‐COR Biosciences (1:10,000; Lincoln, NE, USA) were used as secondary antibodies and signals were detected and quantified using an Odyssey Fc imaging system and the Image Studio software (LI‐COR Biosciences).

### 4.10 Treatment of Organoid‐Induced Xenografts

Six‐week‐old male and female athymic nude‐Foxn1nu mice (Envigo) were inoculated subcutaneously with 1 × 10^6^ 143B‐DXR osteosarcoma organoids suspended in BM and mixed 1:1 with BD Matrigel Matrix (Corning), previously diluted 1:1 in culture medium. Once tumors reached approximately 50–100 mm^3^, mice were randomly assigned (*n* = 7 per group) to receive intravenous (i.v.) tail injections or oral gavage (o.g.) administration of: (1) 5% DMSO in corn oil (o.g. vehicle) every day plus saline (i.v. vehicle; B. Braun, Melsungen, Germany) twice a week; (2) 40 mg/kg dasatinib (o.g.) every day; (3) 4 mg/kg doxorubicin (i.v.) twice a week; (4) 40 mg/kg dasatinib (o.g.) every day plus 4 mg/kg doxorubicin (i.v.) twice a week. Tumor size was measured throughout the experiment with a caliper twice a week, and tumor volume was determined using the equation (4/3 × *π* × [*L*/2 × *W*/2 × *H*])/2, where *L* is tumor length, *W* is tumor width, and *H* is tumor height; systemic toxicity was evaluated by recording animal weight twice a week. Values in each xenograft group were averaged. To study the effect of various drugs on tumor growth, relative tumor volume (RTV) was defined as the difference between the tumor volume at a specific day of measurement (*V*_t_) and the tumor volume at the beginning of the treatment (*V*_0_). Drug efficacy was expressed as the percentage tumor growth inhibition (% TGI), calculated as: TGI (%) = (1 − *T*/*C*) × 100, where *T* is the mean RTV of the treated tumor, and *C* is the mean RTV in the control group on the day of measurement. Mice were sacrificed by cervical dislocation, and tumors were extracted and processed for histological analysis as described above. No criteria were set for including or excluding animals and no animal was excluded from the study. In any case, one mouse from the control series and one mouse from the dasatinib series were sacrificed during the course of experiment due to severe clinical signs induced by tumor growth. Simple manual randomization was used to assign the animals to experimental groups. The experimental data were collected in a nonblinded manner. Histological scoring was analyzed in a blinded manner.

### 4.11 Statistical Analysis

For the in vitro and in vivo experiments, the statistical analyses were performed using the GraphPad Prism software version 10.2.3 (GraphPad Software, Inc). Data are presented as the mean ± standard deviation or SEM as indicated, of at least three independent experiments. Two‐sided Student's *t* or one/two‐way ANOVA Tukey's tests were performed to determine the statistical significance between groups. In comparison with reference groups, the values of *p* < 0.05 were considered statistically significant (ns: no significant; *p < 0.05; **p < 0.01; ***p < 0.001; ****p < 0.0001).

## Author Contributions

**Borja Gallego**: investigation; methodology; data curation; formal analysis; writing – original draft. **Dzohara Murillo, Carmen Huergo, Daniela Corte‐Torres, Andrea Ferreras, Veronica Rey, and Enol Álvarez‐González**: investigation; methodology. **Ivan Fernández‐Vega and Adela A. Fernández‐Velasco**: data curation; formal analysis. **Alejandro Braña and Jose Luis Llorente**: acquisition and provision of samples; methodology. **Mara Santarelli and Benno Schwikowski**: data curation; formal analysis; methodology. **Mónica Alvarez‐Fernández**: conceptualization; methodology; writing – review and editing. **René Rodríguez**: conceptualization; resources; data curation; formal analysis; supervision; funding acquisition; project administration; writing – original draft; writing – review and editing. All authors have read and approved the final manuscript.

## Funding

This work was supported by the Agencia Estatal de Investigación [AEIMICINN/ Fondo Europeo de Desarrollo Regional (FEDER) (grants PID2022‐142020OB‐I00 and PID2019‐106666RB‐I00 to R.R.)], the Instituto de Salud Carlos III (ISC III)/FEDER (grants PT20/00161 and PT23/00077 to I.F.V. and Consorcio CIBERONC—CB16/12/00390), and the Plan de Ciencia Tecnología e Innovación del Principado de Asturias (PCTI)/FEDER (grant IDI/2021/000027 and IDI/2024/000741 to R.R.). B.G. and D.M. were supported by Severo Ochoa predoctoral fellowships (Plan de Ciencia Tecnología e Innovación del Principado de Asturias/FEDER; grants BP‐20–046 and BP‐21–084, respectively). C.H. and A.F. were supported by a predoctoral fellowship from the Fundación Científca de la Asociación Española Contra el Cáncer (AECC). Finally, the authors would like to acknowledge the support from Asociación GALBAN‐Niños con Cancer (grant 2022 to R.R.).

## Ethics Statement

Human samples included in this study were provided by the Principado de Asturias BioBank (PT23/00077) upon obtaining written informed consent from patients. All experimental protocols have been performed in accordance with institutional review board guidelines and with the Declaration of Helsinki and were approved by the Institutional Ethics Committee of the Principado de Asturias (ref: 255/19; date: February 28, 2020). All animal research protocols were carried out in accordance with the institutional guidelines of the University of Oviedo and the EU Directive 2010/63/EU for animal experiments and were approved by the Animal Research Ethical Committee of the University of Oviedo prior to the study (refs: PROAE 34/2019, July 16, 2019; and PROAE 33/2023, November 17, 2023). All mice were maintained under pathogen‐free conditions in the Animal Facility of the University of Oviedo following current regulations.

## Conflicts of Interest

The authors declare no conflicts of interest.

## Supporting information




**Supporting File 1**: mco271134‐sup‐0001‐Video S1.png.

**Supporting File 2**: mco271134‐sup‐0002‐Video S1.MP4

**Supporting File 3**: mco271134‐sup‐0003‐SuppMat.pdf.

## Data Availability

All relevant aggregate data supporting the findings of this study are available within the article. Raw data files obtained from RNA seq and WES analyses have been deposited at the NCBI repositories GEO (reference GSE303294) and SRA (bioproject accession number PRJNA1472804), respectively.
