# Supplementary material for: Advancing in Sarcoma 3D Modeling: Identification of SRC as an Actionable Vulnerability in Doxorubicin‐Resistant Osteosarcoma Organoids
Source: MedComm (2020). 2026 Nov 8;7(11):e71134. doi: 10.1002/mco2.71134 (PMC13744650; doi:10.1002/mco2.71134)
Supplement: Supplementary file 1 [file MCO2-7-e71134-s002.pdf]

ADVANCING IN SARCOMA 3D MODELING: IDENTIFICATION OF SRC AS AN ACTIONABLE VULNERABILITY IN DOXORUBICIN-RESISTANT OSTEOSARCOMA ORGANOIDS

Borja Gallego[1,2], Dzohara Murillo[1,2], Carmen Huergo[1,2], Daniela Corte-Torres[1,3], Andrea Ferreras[1,2], Iván Fernández-Vega[1,2,3,4], Adela A. Fernández-Velasco[1,2,3,4], Verónica Rey[1,2], Enol Álvarez-González[1,2], Alejandro Braña[1,2,5], Jose Luis Llorente[1,2,6,7], Mara Santarelli[8], Benno Schwikowski[8], Mónica Álvarez-Fernández[1,2,9], René Rodríguez[1,2,7,10*]

[1] Instituto de Investigación Sanitaria del Principado de Asturias (ISPA), Oviedo, Spain.
[2] Instituto Universitario de Oncología del Principado de Asturias (IUOPA), Oviedo, Spain.
[3] Biobanco del Principado de Asturias, Oviedo, Spain.
[4] Servicio de Patología, Hospital Universitario Central de Asturias, Oviedo, Spain.
[5] Servicio de Traumatología, Hospital Universitario Central de Asturias, Oviedo, Spain.
[6] Servicio de Otorrinolaringología, Hospital Universitario Central de Asturias, Oviedo, Spain.
[7] CIBER en oncología (CIBERONC), 28029 Madrid, Spain.
[8] Computational Systems Biomedicine Lab, Institut Pasteur, Université Paris Cité, Paris, France.
[9] Centro de Investigación del Cáncer, CSIC-Universidad de Salamanca, Salamanca, Spain.
[10] Centro de Investigación en Nanomateriales y Nanotecnología (CINN), Consejo Superior de Investigaciones Científicas (CSIC), El Entrego, Asturias, Spain

SUPPLEMENTARY INFORMATION

Figure S1

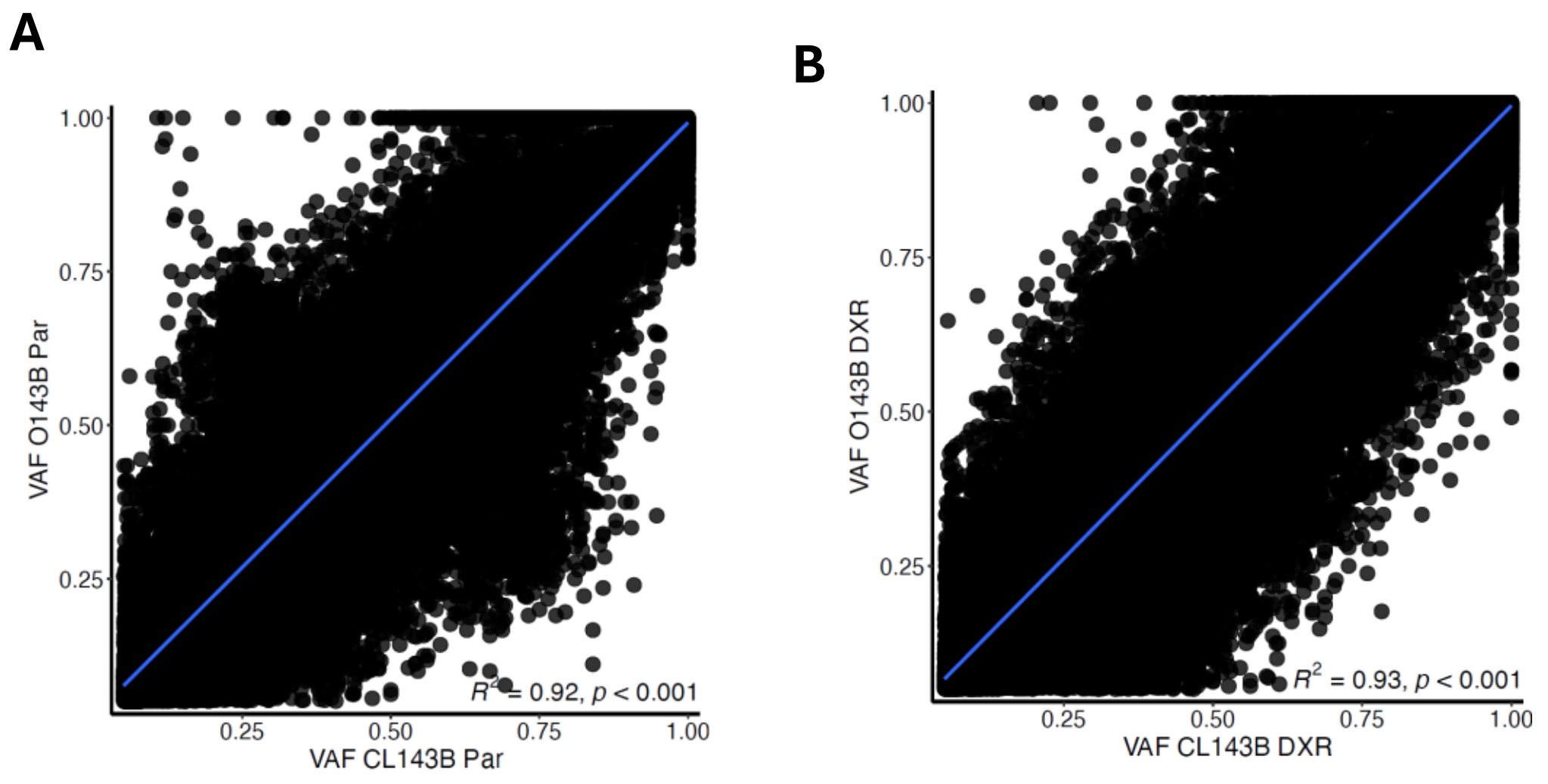

Figure S1. Concordance between WES of cell lines and the correponding xenograft-derived organoids. Correlation analysis of variants shared between the cell line (CL) and organoids (O) of the 143B-Par model (A) and the 143B-DXR model (B). R^2 correlation values and p-values are indicated.

Figure S2

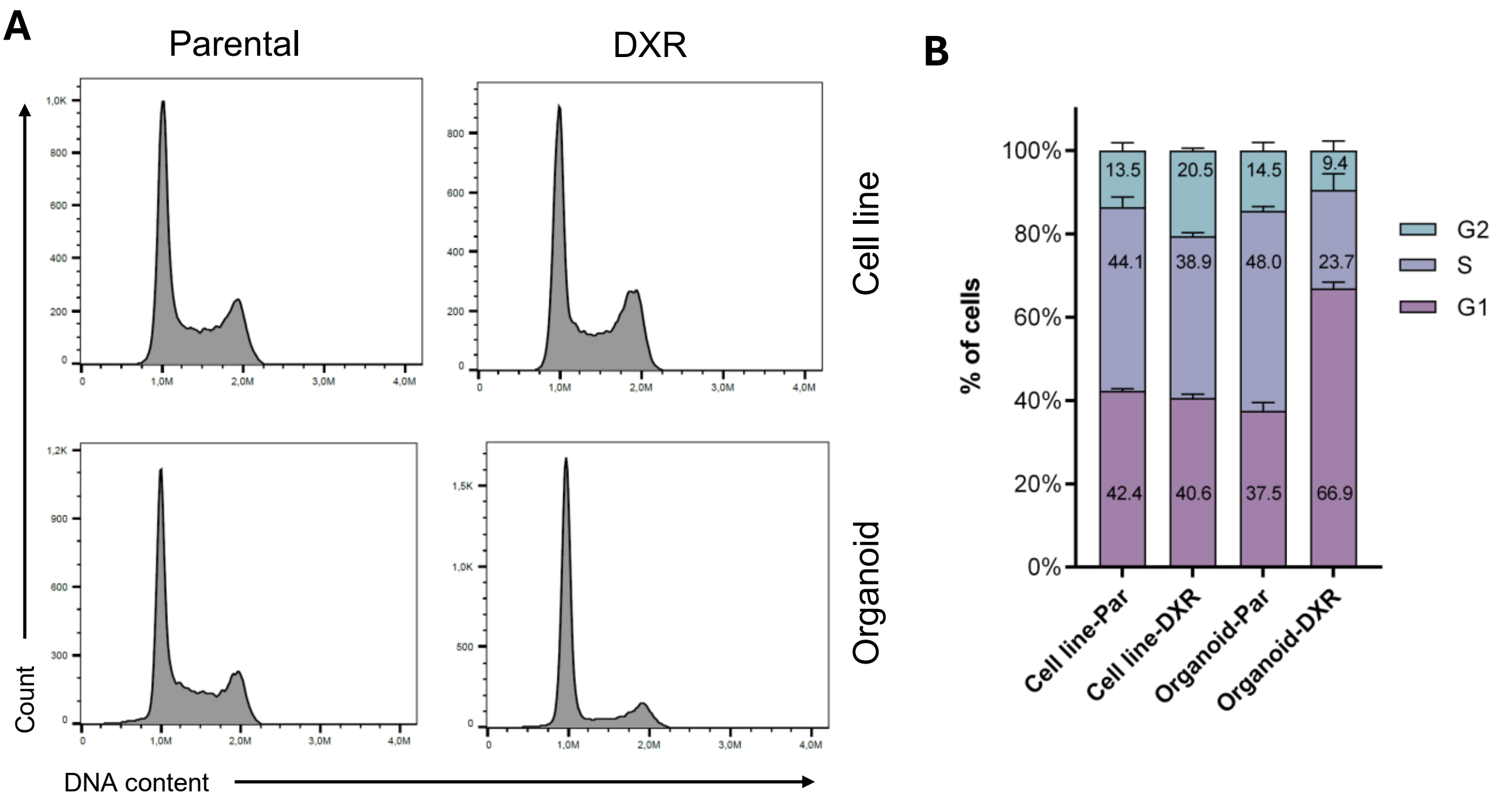

Figure S2. Cell cycle distribution in monolayer cell lines and organoid cultures of the 143B parental and doxorubicin resistant (DXR) models. (A) Representative flow cytometry profiles. (B) Normalized percentage of cells in each cell cycle phase (mean ± standard deviation of three independent experiments).

Figure S3

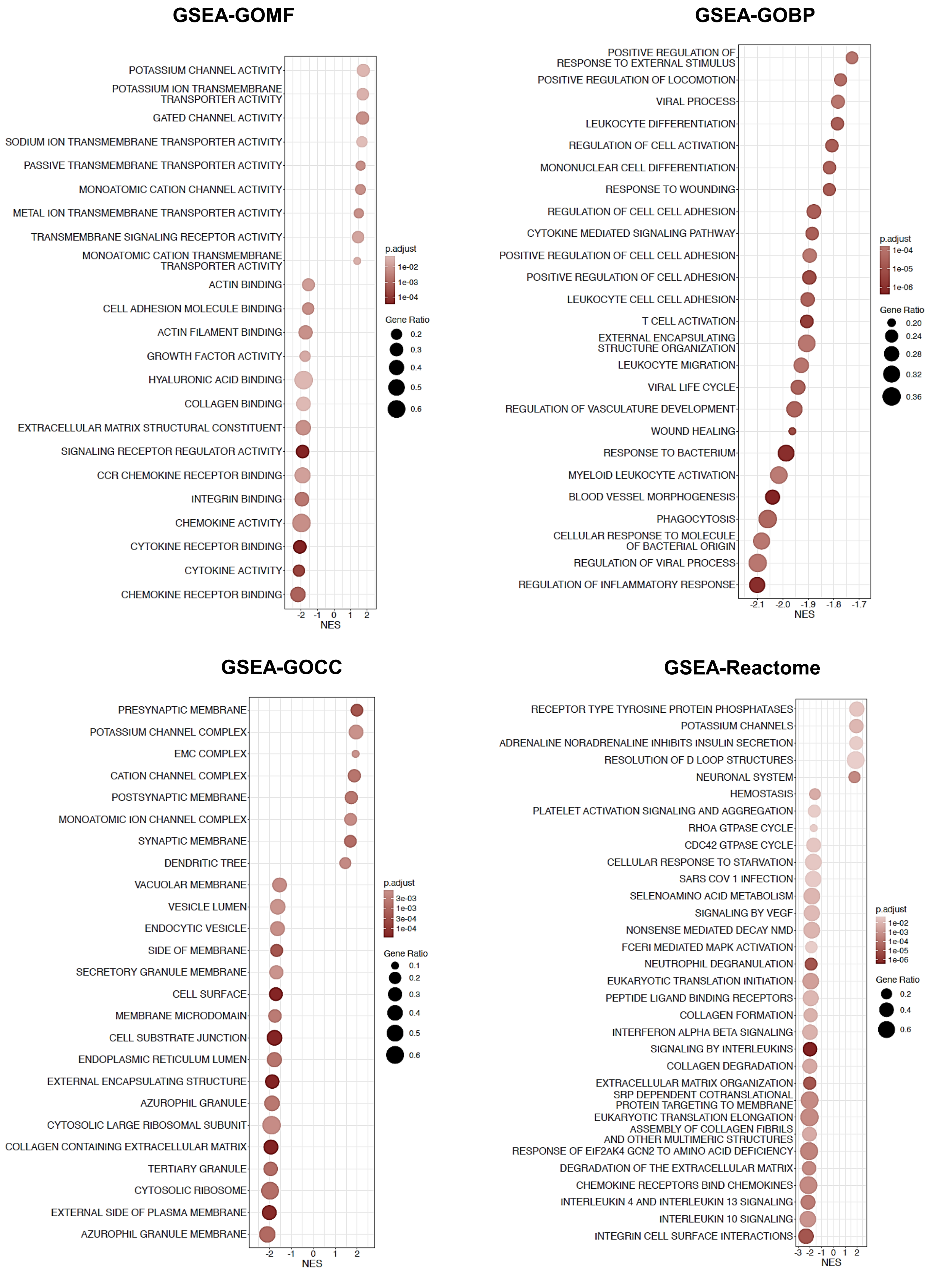

Figure S3. Significantly enriched pathways (GSEA, FDR <0.05) from the GOMF, GOBF, GOCC and Reactome collections in 143B-DXR vs 143B Par organoids.

Figure S4

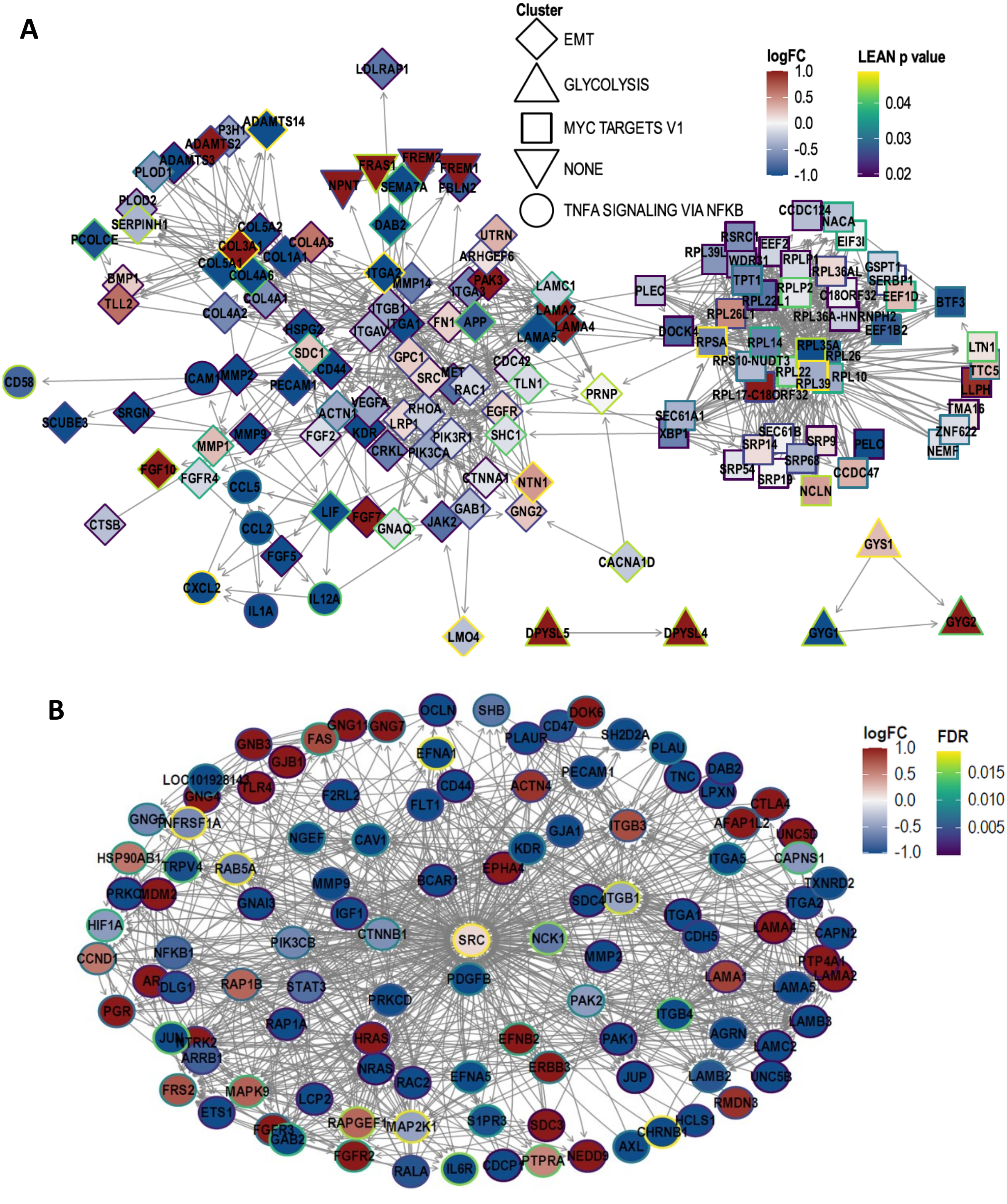

Figure S4. Figure 4. Transcriptomic characterization of the resistant phenotype in 143B-DXR organoids. (A) Complete network representation of the LEAN analysis showed in Figure 4C, including all central nodes with statistically significant aggregate transcriptomic modulation (FDR<0.05). The color of the node indicates the log2FC from the differential expression analysis, while the color of the node border indicates corrected p-value of the aggregate transcriptomic modulation according to LEAN. An unsupervised clustering of this network was performed, and the overrepresented pathway in each group is represented by the node shape. (B) Complete representation of the SRC-centered local network showed in Figure 4E, including all significant nodes. Node color indicates the log2FC and the border color represents the FDR from the differential expression analysis.

Figure S5

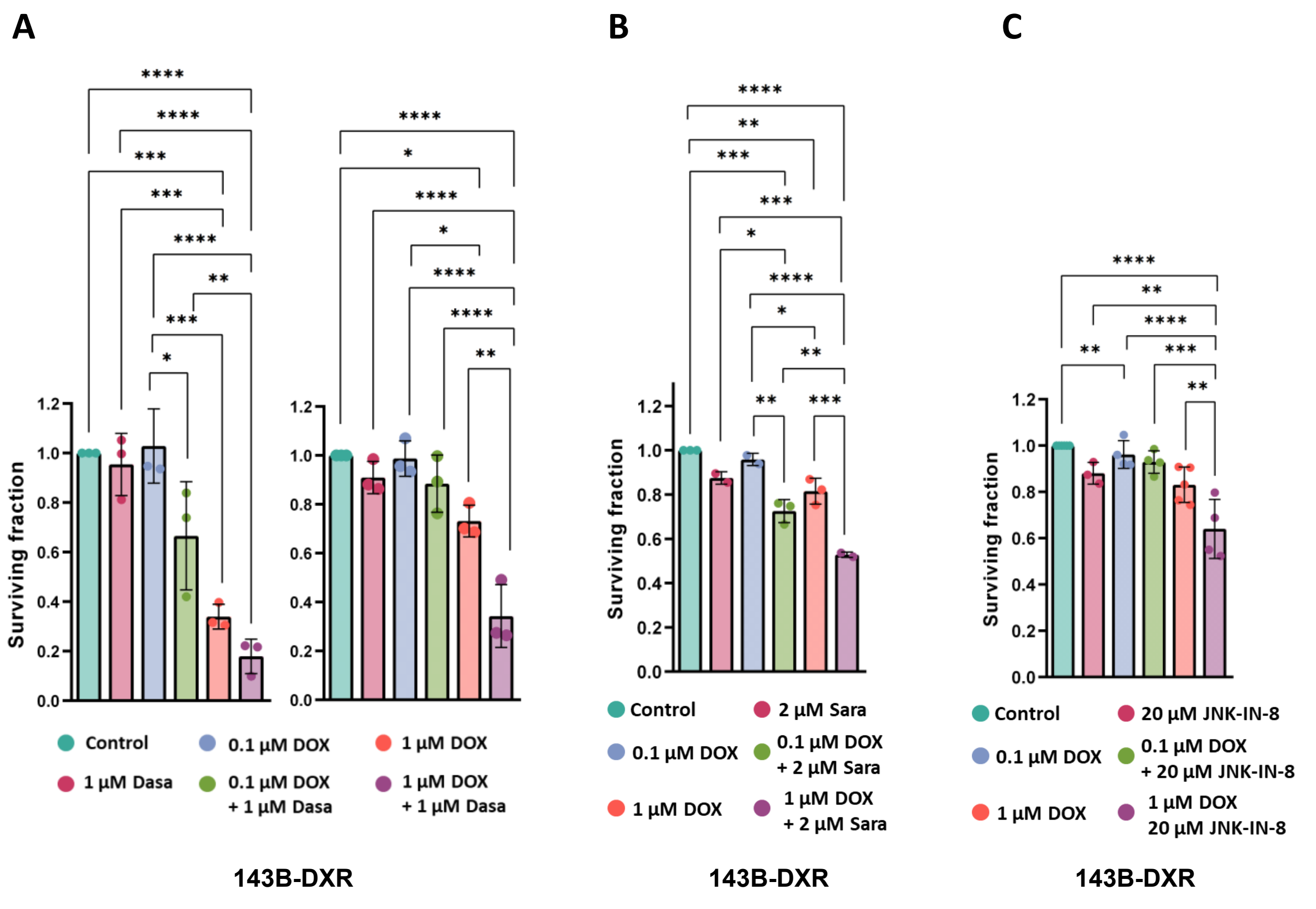

Figure S5. SRC inhibition improves the response to doxorubicin in 143B-DXR organoids. Panels of the cell viability (CellTiterGlo-3D assays) experiments shown in Figure 5 including all significant differences between the treatments. One-way ANOVA tests. *: $p < 0.05$; **: $p < 0.01$; ***: $p < 0.001$; ****: $p < 0.0001$.

Figure S6

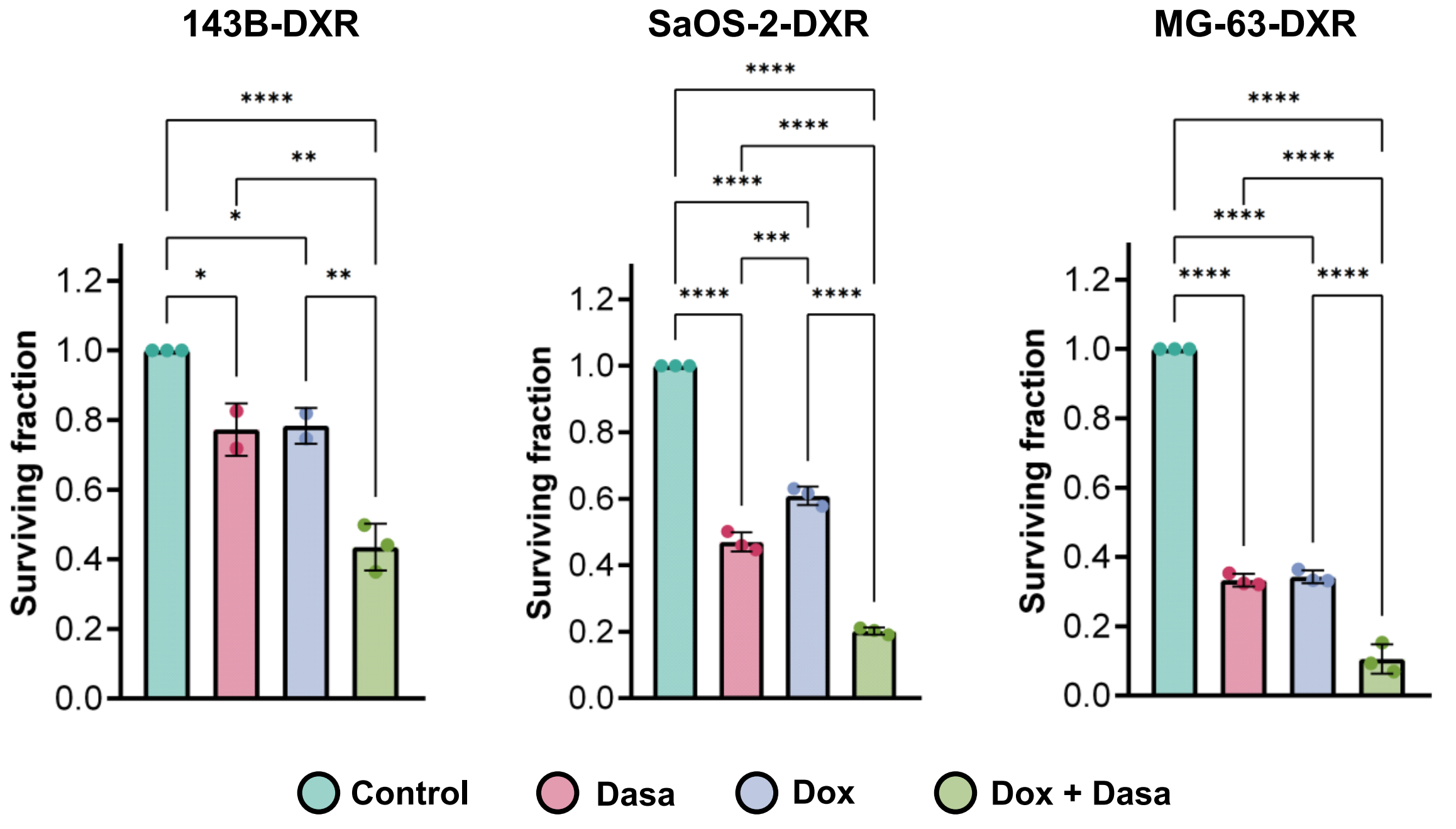

Figure S6. SRC inhibition improves the response to doxorubicin in 2D doxorubicin-resistant cell lines. Cell viability (WST1 assays) measured after treatment of the indicated doxorubicin-resistant (DXR) osteosarcoma cell lines with DMSO (Control), 1 µM Dasatinib (Dasa), 1 µM Doxorubicin (Dox) or the combination of both drugs for 72 hours. Error bars represent standard deviation of three independent experiments, and asterisks indicate statistically significant differences: p < 0,05; **: p < 0,01; ***: p < 0,001; ****: p < 0,0001.

Figure S7

A

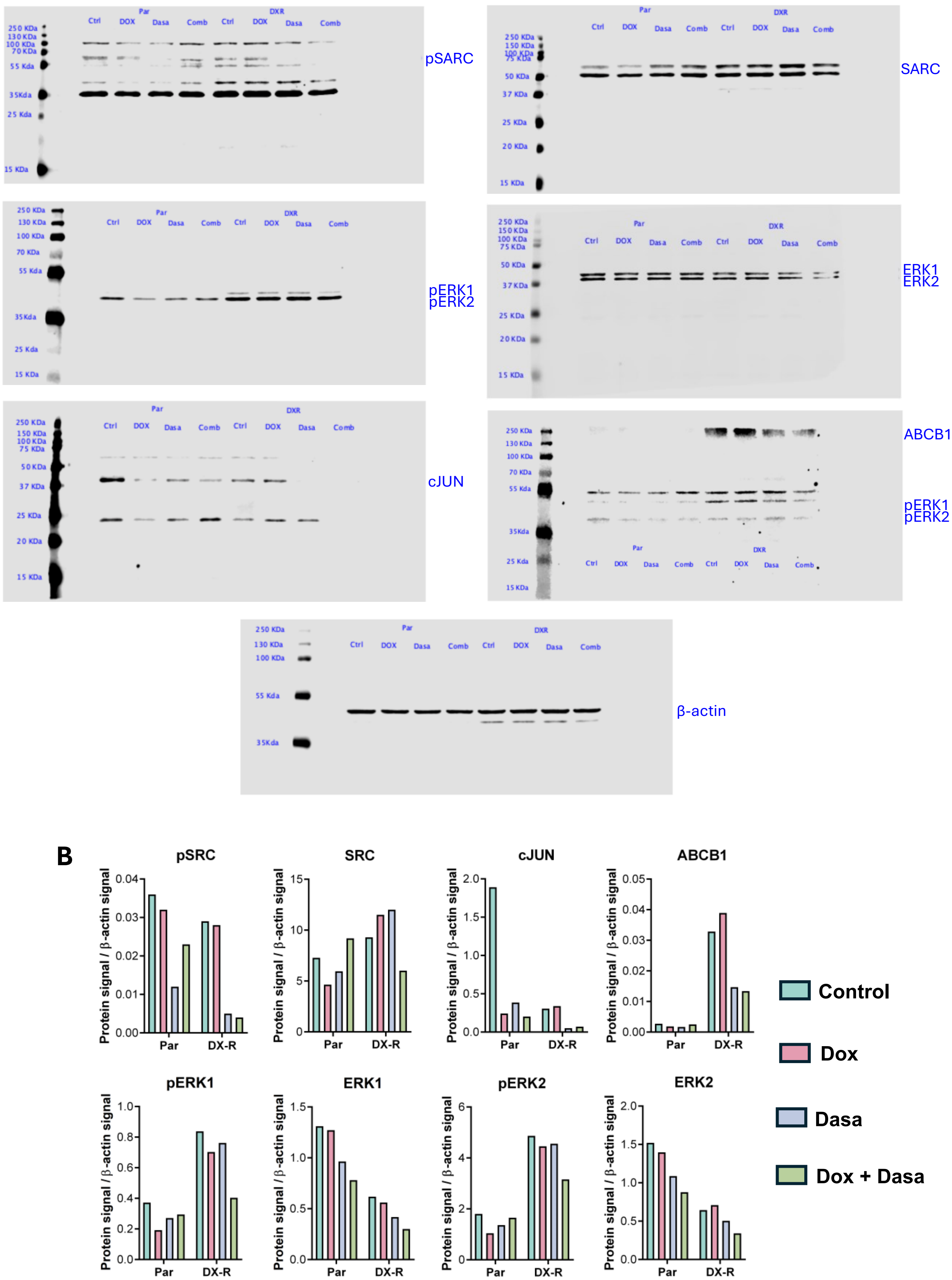

Figure S7. (A) Whole Western Blottings from figures 5C. (B) Quantification **(**Protein levels normalized to β-actin) of Western blots of figure 5C.

Figure S8

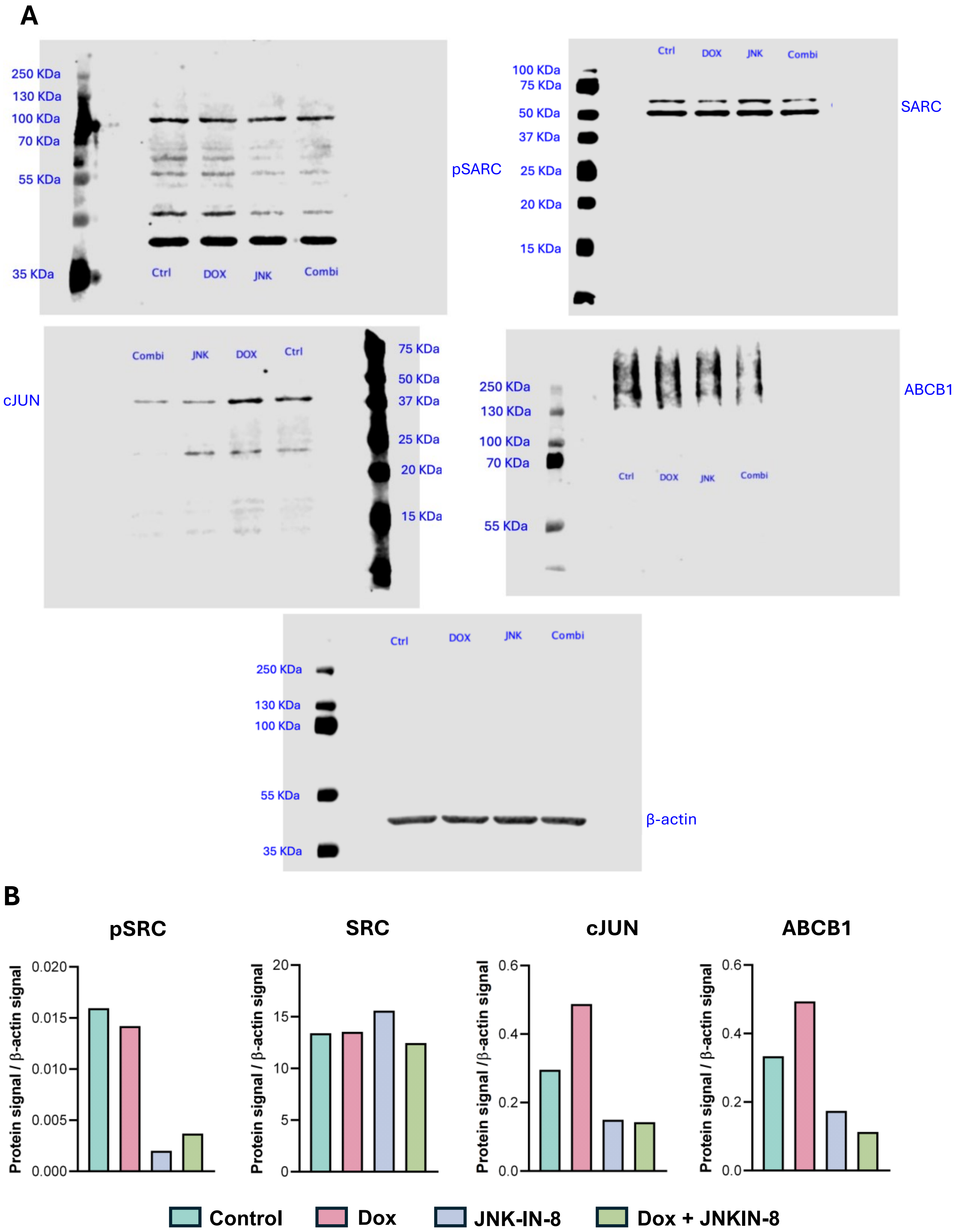

Figure S8. (A) Whole Western Blottings from figures 5E. (B) Quantification **(**Protein levels normalized to β-actin) of Western blots of figure 5E.

Table S1. Culture of patient-derived organoids

ID	Diagnosis	Disgregation Method	Culture Medium	3D Structures	Passages
**LPS-07**	Liposarcoma	Gentle MACs Standard Protocol	BM + 1x B27 + 1.25 mM NAC + 10 mM NIC + 0.05 µg/mL EGF + 0.01 µg/mL FGF10 + 0.005 µg/mL FGF2 + 0.1 µg/mL RSPO1 + 3 µM CHIR 99021 + 1 µM Forskolin (**M0**)	Yes	1
**OTB-01**	Osteoblastoma	Gentle MACs Standard Protocol	M0	Yes	1
**SARC-22**	Soft Tissue Sarcoma	Gentle MACs Standard Protocol	M0	Yes	1
**LPS-08**	Liposarcoma	Gentle MACs Standard Protocol	1. M0<br>2. M0 + 0.5 µM A83-01<br>3. M0 + 2 ng/mL BMP-2<br>4. M0 + 2 ng/mL BMP-2 + 0.5 µM A83-01	Yes	1
**LMS-01**	Leiomyosarcoma	1 mg/mL Collagenase IV (2 h)	M0 + 20 ng/mL IL6 (**M1**)	Yes	0
**LPS-09**	Liposarcoma	2 mg/mL Collagenase IV + 2.25 U/mL Dispase II (45 min)	M1	Yes	0
**CDS-19**	Dedifferentiated Chondrosarcoma	2 mg/mL Collagenase IV + 2.25 U/mL Dispase II (1.5 h)	M1	No	0
**SARC-23**	Myxofibrosarcoma	2 mg/mL Collagenase IV + 2.25 U/mL Dispase II (1.5 h)	M1	No	0
**CDS-20**	Extraskeletal Myxoid Chondrosarcoma	2 mg/mL Collagenase IV + 2.25 U/mL Dispase II (1 h)	M1	No	0
**LMS-02**	Leiomyosarcoma	2 mg/mL Collagenase IV + 2.25 U/mL Dispase II (1.5 h)	M1	No	0
**CDS-21**	Central Chondrosarcoma	5 mg/mL Collagenase IV + 2.25 U/mL Dispase II (1 h)	M1	No	0
**CDS-22**	Extraskeletal Myxoid Chondrosarcoma	5 mg/mL Collagenase IV + 2.25 U/mL Dispase II + 0.05 % Trypsin (1 h)	M1	No	0
**CDS-23**	Secondary Peripheral Chondrosarcoma	5 mg/mL Collagenase IV + 2.25 U/mL Dispase II (1 h)	1. M1<br>2. M1 + 20 ng/mL IL8 (**M2**)<br>3. M1 + 50% MSCs conditioned media	No	0
**CDS-24**	Chondrosarcoma	5 mg/mL Collagenase IV + 2.25 U/mL Dispase II (1 h)	1. M2<br>2. M2 + 50 % MSCs conditioned media	No	0
**SARC-25**	Osteosarcoma	5 mg/mL Collagenase IV + 2.25 U/mL Dispase II (1 h)	M2	No	0
**SARC-26**	Rhabdomyosarcoma	5 mg/mL Collagenase IV + 2.25 U/mL Dispase II (1 h)	1. M2<br>2. M2 + 2 ng/mL PDGFa<br>3. M2 + 2ng/mL PDGF-a +2ng/mL PDGF-b<br>4. M2 + 2ng/mL TGF-B	No	0
**SARC-24**	Undifferentiated Pleomorphic Sarcoma	5 mg/mL Collagenase IV + 2.25 U/mL Dispase II (1 h)	M2	Yes	0
**OST-5**	Osteoblastic Osteosarcoma	5 mg/mL Collagenase IV + 2.25 U/mL Dispase II (1 h)	M2	No	0
**OST-6**	Dedifferentiated High-grade Osteosarcoma	5 mg/mL Collagenase IV + 2.25 U/mL Dispase II (1 h)	M2	No	0

BM: Basal Medium (AdDMEM/F12 supplemented with 1 % HEPES, 1 % GlutaMAX and 1 % Penicillin-Streptomycin. NAC: N-Acetyl-L-Cystein. NIC: Nicotinamide

Table S2. Culture of xenograft-derived organoids

Xenograft Model	Subtype	Model Type	Inoculation Site	Culture Medium	3D Structures	Passages	Biobanked
**143B**	Osteosarcoma	Cell Line	SC	1. M2<br>2. M2 + 10ng/mL PDGF-a +10ng/mL PDGF-b<br>3. M2 + 10ng/mL TGF-B<br>4. M2 + 10nM XMU-MP-1<br>5. M2 + 20ng/mL Pam3Cys	Yes	>20	Yes
**143B**	Osteosarcoma	Cell Line	IB	M2	Yes	8	Yes
**143B-DXR**	Osteosarcoma	Cell Line	SC	M2	Yes	>20	Yes
**MG-63**	Osteosarcoma	Cell Line	SC	M2	Yes	3	Yes
**SaOS-2**	Osteosarcoma	Cell Line	SC	M2	No	0	No
**OS833**	Osteosarcoma	PDXCL	SC	M2	Yes	2	No
**OS742**	Osteosarcoma	PDXCL	SC	M2	No	0	No
**OS186**	Osteosarcoma	PDXCL	SC	M2	No	0	No
**TX-SARC-24**	UPS	PDX	SC	M2	Yes	0	No
**A4573**	Ewing Sarcoma	Cell Line	SC	M2	No	0	No
**A4573**	Ewing Sarcoma	Cell Line	IB	M2	Yes	0	No
**T-CDS-17#3**	Chondrosarcoma	PDXCL	IB	M2	Yes	4	Yes
**T-CDS-17#3**	Chondrosarcoma	PDXCL	SC	M2	Yes	8	Yes
**T-CDS-17#4**	Chondrosarcoma	PDXCL	SC	M2	Yes	3	Yes

SC: subcutaneous. IB: intrabone. PDXCL: patient derived xenograft-cell line. PDX: patient derived xenograft. UPS: undifferentiated pleomorphic sarcoma

Table S3. Short Tandem Repeat analysis for the 143B organoid lines.

locus name	143B - PAR	143B - DXR	143B cell line CRL-8303*
**D7S820**	11,12	11,12	11,12
**CSF1PO**	12,12	12,12	12,12
**TH01**	6,6	6,6	6,6
**D13S317**	12,13	12,12	12,12
**D16S539**	10,13	10,13	10,13
**VWA**	18,18	18,18	18,18
**TPOX**	11,11	11,11	11,11
**AM**	X,X	X,X	X,X
**DS5818**	13,13	13,13	13,13

* CRL-8303. Profile retrieved from the ATCC STR profile database
